# Uncovering Cellular Interactome Drivers of Immune Checkpoint Inhibitor Response in Advanced Melanoma Patients

**DOI:** 10.1007/s12195-025-00857-y

**Published:** 2025-09-08

**Authors:** Shay Ladd, Anne M. Talkington, Mary O’Sullivan, Robert W. Barnes, Remziye E. Wessel, Gabriel F. Hanson, Sepideh Dolatshahi

**Affiliations:** 1 Department of Biomedical Engineering, University of Virginia (UVA) School of Medicine, Charlottesville, VA 22908 USA; 2 University of Virginia Comprehensive Cancer Center, Charlottesville, VA 22908 USA; 3 Beirne B. Carter Center for Immunology Research, UVA School of Medicine, Charlottesville, VA 22908 USA; 4 Division of Pharmacokinetics, Pharmacodynamics, and Systems Pharmacology, Department of Pharmaceutical Sciences, University at Buffalo, SUNY, Buffalo, NY 14214 USA

**Keywords:** Melanoma, Cell interactions, Immunotherapy, Network models

## Abstract

**Purpose:**

Despite the success of immune checkpoint inhibitors (ICIs) that target immunosuppressive interactions, treatment resistance remains a major clinical challenge. The tumor microenvironment is comprised of tumor, immune, and stromal cell types that communicate through secreted and cell surface proteins. This can be represented by a weighted, directed network where pairs of cell types communicate via multiple ligand-receptor interactions with varying strengths. Identifying interaction network motifs that are linked with outcome or evolve pre- to post-ICI presents a rational framework to identify combination therapeutic targets.

**Methods:**

Interaction inference was performed on publicly available single-cell RNA-sequencing data from melanoma patients. The constructed patient-specific networks were input to multivariate statistical learning approaches to identify network motifs that predicted response pre-treatment and that shifted pre- to post-treatment. Relevance of interactions was validated by (1) differential expression of related pathways in single cell RNA sequencing (scRNA-seq) data, (2) survival associations in an independent bulk RNA-seq dataset, and (3) repeated analyses of scRNA-seq data in a second cohort.

**Results:**

Immune-immune interactions with roles in T cell activation, chemotaxis, and adhesion were upregulated in patients who respond to therapy pre-treatment. Related pathways were perturbed in involved immune cells and expression of these genes was associated with improved survival. The interactome also distinguished pre- and post-treatment biopsies with high accuracy despite no significant differences in individual interactions. Analysis in the validation dataset with mixed responses pre-treatment recapitulated results from the discovery analyses.

**Conclusion:**

Unbiased analysis of interaction networks and their evolution is a powerful framework to guide prognostic indicators and novel combination targets to improve patient outcomes.

**Supplementary Information:**

The online version contains supplementary material available at 10.1007/s12195-025-00857-y.

## Introduction

An estimated 100,000 people in the United States are diagnosed with melanoma annually and more than 8,000 melanoma deaths are projected to occur in 2025 [1]. In the last 15 years, standard-of-care immunotherapies for metastatic melanoma have doubled survival from 18% in 2009 to 35% in 2020, but their efficacy remains limited as many patients will have tumor recurrence regardless of initial therapeutic response [1–3]. Common immunotherapies include immune checkpoint inhibitors (ICIs) that disrupt signaling by checkpoint molecules such as cytotoxic T-lymphocyte-associated protein-4 (CTLA-4), programmed cell death protein-1 (PD-1), or programmed cell death-ligand 1 (PD-L1) [4]. In combination, PD-1 blockade (nivolumab, pembrolizumab) and anti-CTLA-4 (ipilimumab) have achieved a 1 year survival rate of 85% as opposed to 63–69% for anti-PD-1 alone in metastatic melanoma, underscoring the potential for combination immunotherapies to improve patient outcomes [5].

The tumor microenvironment (TME) is comprised of various tumor, immune, and stromal cell types that communicate through a combination of secreted and cell surface proteins. This communication can be represented by a weighted and directed network, where each pair of cell types communicates via multiple ligand-receptor (LR) interactions with varying strengths. In this cell interaction network, cell types are represented as nodes and an edge is drawn between them weighted by an interaction score derived from the cells’ expression of corresponding ligands and receptors. Communication is directed from the cell type expressing the ligand to the cell type expressing the receptor. Leveraging this interaction information has the potential to improve response to ICIs, which themselves target a LR interaction. Prior work has identified TME-based biomarkers associated with response in melanoma and implicated immune cell interactions as key drivers of ICI response [6–8]. For example, tumor major histocompatibility complex (MHC)-I and -II expression prior to treatment with anti-CTLA-4 and anti-PD-1 ICIs is associated with improved response [6, 9, 10]. CD8 + T cell-induced interferon gamma (IFN-γ) signaling with tumor cells is also positively associated with response [8]. In depth profiling of immune cell populations in the TME of pre-treatment melanoma samples has shown that response to ICI correlates with increased CD8 + T cell counts and natural killer (NK) cell counts, along with decreased macrophage cell counts [7]. Together, these findings point to a key role for immune interactions in driving ICI response and underscore the need for a holistic and unbiased approach to identify interactions and network motifs that contribute to non-response. Understanding the aggregate of potential intercellular communication mechanisms also lays the foundation for suggesting alternative targeting strategies based on immunosuppressive interactions that arise or immunostimulatory interactions that disappear in the TME following immune checkpoint blockade.

Communication network inference, the process of determining the system of nodes and edges representative of a suite of communication pathways, is an established strategy to determine gene regulatory networks within cells to infer signaling processes [11–16]. More recently, network inference strategies have been used to infer communication between cells [17–25]. Here, we examine network remodeling in two pre- and post-treatment cohorts of patients receiving ICI targeting the PD-1/PD-L1 checkpoint or CTLA-4. Taking advantage of the high resolution of single cell RNA-sequencing (scRNA-seq) data in independent discovery and validation cohorts, we uncover the sets of cell types and their communication patterns that predispose a TME to ICI treatment response. These cellular communication networks likely contribute to cell state transitions in various immune cell populations and present complimentary insights to those drawn from tracking cellular state or TME composition over time. We also identify interaction network remodeling that occurs from pre- to post- treatment in both responders (R) and non-responders (NR), and protein-protein communication mechanisms associated with failure to respond to ICI. These signals suggest mechanisms of resistance for future investigation. Understanding these processes enables improvements in current strategies and suggests complementary strategies as we investigate new targetable regulators in the immunosuppressive TME.

## Methods

### Single-Cell RNA-sequencing Data Processing and Cell Type Reclassification

scRNA-seq data were obtained from published studies conducted by Sade-Feldman et al. [26] (discovery dataset; GSE120575) and Jerby-Arnon et al. [27] (validation dataset; GSE115978) (Table 1). The discovery dataset consisted of single cells from 48 biopsies—9 R pre-ICI, 8 R post-ICI, 10 NR pre-ICI, and 21 NR post-ICI—from 32 patients, with 12 patients involved in longitudinal data collection. Of these 12, two patients had two biopsies taken post-treatment. Response was classified on a per-lesion, not per-patient basis, so one additional patient had mismatched lesion response pre- and post-treatment. This left 6 paired NR and 3 paired R samples for matched analyses. Patients in this study all received the anti-PD-1 therapeutic pembrolizumab, with a subset of 8 patients receiving anti-CTLA-4 as a combination therapy.

**Table 1:** Melanoma patient characteristics

Pre/post, response designation^a^	Cohort 1: Sade-Feldman et al. (n = 48) [26]					Cohort 2: Jerby-Arnon et al. (n = 32) [27]			
	Pre R (n = 9)	Pre NR (n = 10)	Post R (n = 8)	Post NR (n = 21)	Total	Pre (R/NR) (n = 16)	Post R (n = 1)	Post NR (n = 15)	Total
Biologic sex, Female	2 (22.2%)	4 (40%)	2 (25%)	8 (38.1%)	16 (33.3%)	5 (31.3%)	0 (0%)	5 (33.3%)	10 (31.3%)
Age [years]: mean +/− std (range)	66.44 ± 10.68 (49–79)	64.50 ± 15.04 (29–83)	53.75 ± 17.54 (29–75)	64.67 ± 11.72 (33–83)	63.15 ± 13.62 (29–83)	67.44 ± 14.02 (37–86)	81.00 ± 0.00 (81–81)	67.93 ± 9.69 (47–83)	68.41 ± 10.69 (37–86)
Treatment (anti-PD1/anti-CTLA4/anti-PD1 + anti-CTLA4)	4/0/5	8/0/2	4/0/4	16/0/5	32/0/16	Unknown	0/1/0	2/4/9	2/5/9
Site of biopsy (skin/lymph node/other)	4/5/0	6/1/3	4/3/1	11/4/6	25/13/10	5/9/2	1/0/0	7/3/5	13/12/7
Primary tumors	Not available for Sade-Feldman et al.					4 (25%)	0 (0%)	0 (0%)	4 (12.5%)

*std* standard deviation

^a^Number (%) unless otherwise specified

The validation dataset contains 32 biopsies (16 pre-ICI with unknown response, 1 R post-ICI, 15 NR post-ICI). We removed the only responding post-ICI patient to unambiguously classify post-treatment as NR in this dataset. The patients in this study received the PD-1/PD-L1 blockades nivolumab and pembrolizumab alone or in combination with the CTLA-4 blockades ipilimumab and tremelimumab. Four patients received only CTLA-4 blockade. Detailed patient and biopsy classification is available from the original sources [26, 27].

Data quality control and cell type re-identification were performed on the transcript per million (TPM) matrices for both datasets in R v4.3.1 using the Seurat v5.1.0 package [28]. In the discovery dataset, low quality or dying cells with > 2% mitochondrial genes, low-quality or empty cells with < 500 unique genes, and likely doublets with > 5000 unique genes were removed. For the validation dataset, the TPM matrix had already been filtered to exclude cells with expression of mitochondrial genes and additional filtering removed cells with < 500 unique genes or > 7500 unique genes. Thresholds for filtering were determined based on literature and quality control plots (Fig. S1a, b) [29]. Data was log normalized using the log1p method of NormalizeData. For visualization purposes, principal component analysis (PCA) was performed with RunPCA with 50 components, and an elbow plot was produced to identify the appropriate number of components. Finally, uniform manifold approximation and projection (UMAP) reduction of the cells was performed with 20 dimensions and clusters were defined by K nearest-neighbor (kNN) algorithm, resulting in 21 clusters corresponding to 10 distinct cell types. Differentially expressed genes identified using the limma package and expression of canonical cell type markers were used to assign cell type identities to each of the clusters (Table S1, Fig. S1c, d) [30]. In the validation dataset, cells identified as tumor cells were removed for further analysis of immune cells only. Total cell counts after all pre-processing are provided on a per-patient basis for each dataset (Supplemental Data 1).

To enable granular functional characterization of T cells and myeloid cells, further subgrouping of these populations was performed in Seurat v5.1.0. For the T cell subsets, groups identified as regulatory T cells (Tregs), CD8 + T, CD4 + T, or γδ T cells were isolated, and the 2000 most variable features were identified. Data was then scaled, and PCA was conducted with 50 components. A kNN graph was constructed using the top 20 PCs and used to identify cell clusters. To identify differentially expressed genes, the limma package was used [30]. Limma applies linear modeling and empirical Bayes moderation to estimate expression differences between groups. Differentially expressed genes and canonical markers were used to identify T cell subclusters (Table S2, Fig. S1e). Similarly, myeloid subclusters were identified by isolating the myeloid cells and running the analysis detailed above (Table S2, Fig. S1f).

### Network Inference Using LIANA

For interaction network analyses, we consider each cell type in the TME as a node. An edge connecting the nodes emerges when a ligand on one cell type interacts with its corresponding receptor on another cell type. The weight of the edges corresponds to the strength of the interaction between the LR pair on the cell types of interest. To infer these edges, we used the LIgand-receptor ANalysis frAmework (LIANA), a wrapper which implements multiple LR databases and inference methods to infer and prioritize interactions which appear in a consensus of method outputs [31]. This use of the consensus serves as a built-in validation method as it prioritizes the detected interactions found to be significant across multiple methodologies.

LIANA inference was performed in both the discovery and validation datasets on a per-biopsy basis. We selected the “Consensus” resource which is composed of 4701 known ligand receptor interactions from the CellPhoneDB, CellChat, ICELLNET, connectomeDB2020, and CellTalkDB resources [19, 23, 32–34]. Five inference methods were implemented through the LIANA framework: NATMI, Connectome, log fold change (LogFC), SCA, and CellPhoneDB. Within each method, the resulting inferred interactions were ranked based on their respective scores with 1 being the most significant. These results were then aggregated on a per biopsy basis to get the mean rank, which is the average rank of a given interaction across all five methods. A high ranking (low numeric value) indicates high confidence in the inferred interaction. These mean rank values served as the input for our supervised statistical learning methods.

### Interaction Frequency Calculations

To assess the diversity of communication events occurring between cell type pairs, we computed the number of distinct interactions detected between all classified cell types per biopsy. First, a filter was applied to look at only interactions that are robustly and highly ranked across methods (aggregate rank, a proxy for p-value, of ≤ 0.1) [31]. The data frame was then reformatted such that each row represents a unique LR interaction sorted and grouped by the interaction’s sender-receiver cell type pair. The number of rows per cell pair was counted and the number of inferred interactions was normalized by dividing by the total number of detected interactions in each patient. For sender-receiver cell pairs with no detected interactions, a proportion value of 0 was assigned.

### Imputation of Missing Interaction Values

Imputation of missing values was performed to prepare the mean rank matrix for downstream multivariate statistical analysis. Because PLSDA/R models require a complete matrix, and the LIANA output is inherently sparse, imputation was applied to enable downstream analysis. Imputation is a commonly used approach to mitigate zero-dropout in the context of cell-cell communication inference [35, 36]. Three candidate methods including Random Forest, kNN, and Multiple Imputation by Chained Equations, Predictive Mean Matching (MICE PMM) were considered [37–39]. Each method was tested on the pre-ICI mean rank matrix of the discovery dataset, filtered to retain only interactions with known values in at least 70% of samples. Removing highly sparse interactions supports more reliable imputation and better preserves biological heterogeneity.

To assess imputation quality, the root mean squared error (RMSE) was calculated by first filtering to the subset of interactions that had complete data for all samples. Then, to mimic the missingness observed in the 70% thresholded dataset, an equivalent proportion of values were randomly masked in the complete subset, enabling direct comparison of imputed values to ground truth. The RMSE was scaled by the standard deviation of the ground truth values to produce a normalized RMSE (NRMSE). An NRMSE of 1 indicates that the prediction error is comparable to the inherent variability in the data [40]. Values below 1 suggest that the model captures signal beyond baseline noise and performs better than a naive mean-based prediction. NRMSE is widely used to evaluate model performance in biologically meaningful contexts [41–43].

Non-parametric Random-Forest imputation had the lowest NRMSE of these methods for our data when applied to the transposed mean rank matrix, with interactions as rows (Table S3). This finding aligns with previous work demonstrating that Random Forest tends to outperform alternative imputation strategies when applied to large, complex biological datasets [44, 45]. As a result, Random Forest was selected as the imputation approach, implemented using the missForest function in R [37, 44]. The function parameters were as follows: maximum number of iterations = 20, number of trees per forest = 500, number of variables sampled at each branch = square root of the total number of variables, rounded down, seed = 123.

Although a 0.7 threshold was initially used to select the optimal imputation method, further threshold optimization was performed for each comparison’s mean rank matrix using the NRMSE metric, to refine filtering for optimal imputation performance. A coarse sweep from 0 to 1 at intervals of 0.1 was first conducted to identify the threshold yielding the lowest NRMSE. This was followed by a finer sweep at intervals of 0.01 within ± 0.05 of the best-performing coarse threshold. The resulting optimal threshold for filtering sparse interactions varied for each comparison; in all cases the threshold with the lowest NRMSE was selected (Table S4). Interactions removed by this filtering were excluded from multivariate statistical analyses but were reintroduced in downstream correlative analyses, which are tolerant to missing values. These correlative analyses build upon the results of the multivariate modeling.

### Univariate Statistical Analyses

A Wilcoxon rank-sum test was used to compare the proportion of each cell type normalized to the total cell count per patient between R and NR in the Sade-Feldman dataset. Following protein-protein interaction inference with LIANA, the Wilcoxon test was used to compare the proportion of pairwise intercellular interactions in pre-treatment samples from R and NR in the discovery dataset, normalized to the total number of interactions inferred per patient. To compare cell type proportions and interaction frequencies across R pre- and post-ICI and NR pre- and post-ICI, additional non-parametric tests were performed, such as Wilcoxon rank-sum tests when comparing two groups and Kruskal-Wallis followed by Dunn’s post hoc analysis when comparing more than two groups.

### Multivariate Statistical Analyses

The least absolute shrinkage and selection operator (LASSO) function in MATLAB was used to perform elastic net modeling and identify interactions differentiating groups of interest [46]. Prior to analysis, imputed mean rank values were log10-transformed. We tested alpha values of 0.1, 0.5, and 1 to control elastic net regularization. In all cases, an alpha of 1 was selected based on maximal feature reduction without compromising Cross Validation (CV) accuracy. LASSO modeling was repeated 500 times and features that appeared in ≥ 90% of the models were retained. These features were manually evaluated against literature for biological feasibility (see *Interaction Pruning*). Infeasible interactions were removed and LASSO was rerun iteratively until only biologically plausible interactions remained.

Orthogonalized partial least squares discriminant analysis (OPLSDA) models were built using these LASSO-selected features to identify the interactions that best distinguish R from NR as well as pre- to post-ICI treatment samples. OPLSDA was implemented as described previously and performed in MATLAB 2024b [47]. After building this initial OPLSDA model, LASSO features with Variable Importance in Projection (VIP) scores > 1—indicating above average contribution to the model—were retained and used to build a secondary OPLSDA model. This step further reduced the feature set and mitigated overfitting relative to sample size.

Each model underwent fivefold CV and empirical p-values were calculated by comparing model performance to 1000 randomly permuted null models, providing an additional safeguard against overfitting. These null models were built upon data which have had its labels randomly shuffled.

Top-ranking features were subsequently subjected to univariate comparisons using the Wilcoxon rank-sum test. For visualization, univariate plots display 1/log(mean rank), where smaller rank values indicates greater importance,. To identify covariates of the LASSO-selected features, Pearson correlation analyses were performed on all interactions, including those which were previously filtered out due to missing values. Both LASSO-selected features and their covariates went through an interaction pruning process detailed below, after which, the five features with the strongest correlations (positive or negative) to LASSO-selected features were visualized using a correlation network in Cytoscape V 3.10.0 [48].

### Interaction Pruning

Interactions were excluded if they met any of the following criteria:

1. Subunits of a protein complex that forms only within a single cell, erroneously inferred as intercellular interactions (e.g., G-protein subunits).
2. Interactions between cell types where one or both molecules are localized exclusively to intracellular compartments (e.g., calmodulin interactions within the endoplasmic reticulum).
3. Interactions previously hypothesized as functional but later shown not to occur between any cell type pairs.

All removed interactions and the rationale for exclusion are documented in Supplemental Data 2.

### Pathway Enrichment Analysis

Single-cell pathway analysis was performed using the SCPA R package, which evaluates changes in the multivariate distribution of gene expression within predefined biological pathways [49]. Briefly, pathways were curated by searching MSigDB v2025.1.Hs for gene sets up- and downstream of observed interactions, matched to broader functional categories observed in the co-correlates (Table S5) [50, 51]. Pathways were formatted using the format_pathways function. Cells from pre-treatment samples were then grouped by cell type and treatment response. Data from each group were extracted using the Seurat_extract function. Pathway enrichment was compared between response groups for each cell type using the compare_pathways function. P-value, fold enrichment, and q-value were extracted from each comparison as metrics for the significance, direction, and magnitude of each pathway.

### Survival Analysis

All survival analyses were conducted using Python v3.11.6. Z-scored bulk RNA sequencing data from the Oncology Research Information Exchange Network (ORIEN) AVATAR program were obtained from cBioPortal [52–54]. Patient selection was based on the following criteria: diagnosis of cutaneous melanoma, availability of immuno-oncology drug data (ImmunoOncologyDrug = True), and the presence of RNA sequencing data, with no threshold applied to the z-scored data. Z-scored gene expression data for all genes identified in the pre-treatment R vs. NR analysis were retrieved from the database.

The LR scores for each patient were defined as the mean of z-scored expression values of the ligands and receptors. Only interactions that were enriched in R pre-ICI were included in this analysis and cell type was not considered. Patient clustering was performed using the Ward method, implemented in the clustermap function of the Seaborn library in Python [55]. Overall survival differences between high and low expression groups were assessed using a log-rank test. Kaplan-Meier survival analysis and Cox proportional hazards regression analysis was conducted to compare survival outcomes and determine the 5 year hazard ratios for overall survival between the high and low expression groups, utilizing the lifelines package [56].

## Results

### Immune Cell Frequencies, Immune Cell Interaction Frequencies, and the Abundance of T Cell Subsets Differ Between ICI Responders and Non-responders

**Fig. 1:**
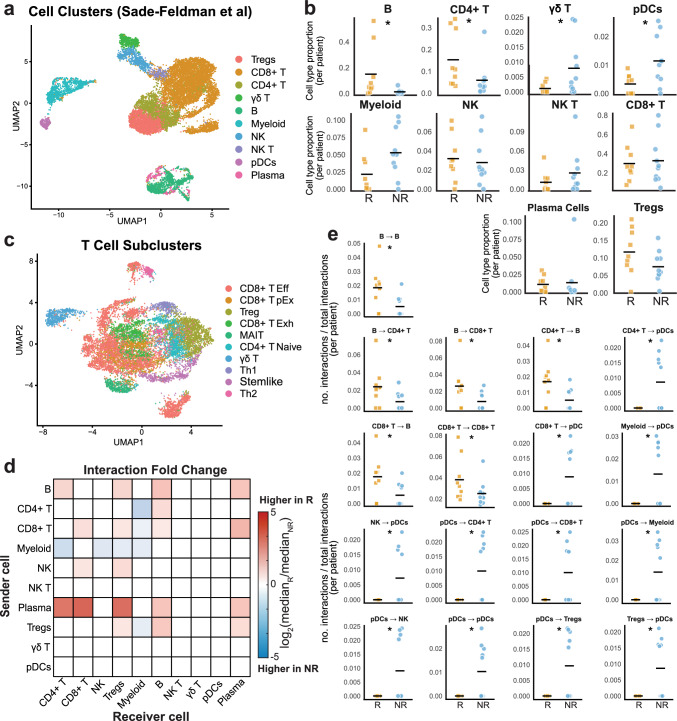
Responders and non-responders exhibit distinct TME profiles of immune cell presence and interaction frequencies. **a** UMAP plot of all immune cells from Sade-Feldman et al. [26]. Treg = regulatory T cells, γδ T = gamma delta T cell, NK = natural killer, pDC = plasmacytoid dendritic cells, plasma = plasma cells. **b** Cell type proportions (the number of each cell type normalized to the total number of cells per patient) compared between treatment responders (R, orange squares) and non-responders (NR, blue circles). *p < 0.05, Wilcoxon rank-sum test. The median is indicated by a black line. **c** UMAP plot showing the re-clustering of all T cells (CD8 T, Treg, CD4 T, γδ T). Treg = regulatory T cells, CD8 + Exh = terminally exhausted CD8 T cells; CD8 + pEx = progenitor exhausted CD8 T cells, CD8 + Eff = effector CD8 T cells, γδ T = gamma delta T cells, Th1 = T helper 1, Th2 = T helper 2, CD4 + Naive = naïve CD4 T cells, MAIT = mucosal associated invariant T cells. **d** Heatmap depicting the fold change of the median number of pairwise cell type interactions between responders and non-responders, with 0.001 added to both median values to prevent division by zero or attempting to take the log of a zero value. The fold change is log2 normalized. Red indicates cells which interact more frequently in responders, and blue indicates cells which interact more frequently in non-responders. **e** Univariate plots showing the comparison of pairwise cell type interaction frequencies between responders (orange squares) and non-responders (blue circles). Only statistically significant comparisons (p < 0.05) are shown. *p < 0.05, Wilcoxon rank-sum test. The median is indicated by a black line

scRNA-seq data from the discovery dataset (Table 1) were used to explore differences in cell type abundance, phenotypes, and the frequency of intercellular interactions between R and NR pre-treatment (Fig. 1). Cell type proportions were compared between the 9 R and 10 NR pre-treatment samples, which revealed that the proportion of B cells and CD4 + T cells were significantly higher in R than in NR (Fig. 1b). The difference in B cells was specifically pronounced in two of the patients (P26 and P35). The proportion of gamma delta (γδ) T cells and plasmacytoid dendritic cells (pDCs) were significantly higher in NR (Fig. 1b). While not statistically significant, myeloid cells were also higher in NR (Fig. 1b). These findings align with the observations from the original Sade-Feldman publication [26]. When focusing on the subset of samples for which matched pre- and post-treatment samples were available, we observed that CD4 + T cell populations decreased in all three R patients and increased in 5 out of 6 NR patients (Fig. S2a).

Further clustering was performed on the T cells identified in the original dataset to identify differences in phenotypes between patient populations (Fig. 1c). The CD8 + T cell population further divided into CD8 + Effectors (CD8 + Eff)*,* terminally exhausted (CD8 + Exh), and progenitor exhausted (CD8 + pEx) subtypes [57, 58]. Both exhausted groups express cytotoxic markers *PRF1* and *GZMB*, as well as exhaustion markers *TOX, PDCD1, LAG3, TIGIT*, and *HAVCR2,* all of which were more highly expressed in the CD8 + Exh group [59, 60]. Many of these CD8 + effector and exhausted clusters also showed high expression of HLA-DR genes. Transient expression of these genes is associated with activation of strong cytotoxic responses and response to neoadjuvant chemotherapy in breast cancer [61]. Clusters of mucosal associated invariant T (MAIT) and γδ T cells are also observed, identified by the expression of *KLRG1*/*TRAV1-2* and *TRDC/TRGC1*, respectively[62–64]. The CD4 + T cell population separated into naïve CD4 (CD4 + Naive), Th1, Th2, and Tregs. These cell types were determined based on canonical and differentially expressed markers (Table S2**, **Fig S1e) A final population, identified as stem-like T cells, was characterized by expression of *CCR7*, *TCF7*, and *FOXP1* with a lack of exhaustion markers (*TIGIT, PDCD1, LAG3, TOX*) [65, 66]. This population contained both CD4 + and CD8 + T cells. R had significantly elevated levels of Th2, CD4 + T naïve, and stem-like populations compared to NR pre-ICI and the proportion of Th2 cells increased pre- to post-ICI in NR (Fig. S2b). In the patients with matched data available, Th2 and naïve CD4 + T cell populations (but not Th1) decreased in all R patients and increased in 5 out of 6 NR (Fig. S2c). Additionally, effector CD8 + T and Th1 populations both increased in 5 out of 6 NR (Fig. S2c). A similar look into the myeloid cell subsets was performed, but extremely low presence of these cells in R samples (both pre- and post-treatment) prevents robust comparisons and interpretations (Fig. S2d).

To investigate the intercellular interaction networks of each sample, we next quantified interaction frequencies between each pair of cell types. Protein-protein interactions were inferred using the LIANA consensus method [31]. The frequency of unique interactions, defined as the total number of interactions between each pair of cell types divided by the total number of interactions inferred for that sample, provides an overview of the interaction landscape in R and NR patient groups and reveals preliminary differences in the interactome without considering the strengths of those interactions. Interactions involving plasma cells, B cells, and CD8 + T cells were higher in R, while those involving myeloid cells were higher in NR (Figs. 1d, S2e). The most significant univariate differences in interaction frequencies were observed in pDC-containing interactions, which were all elevated in NR, however this is likely due to the increased numbers of pDCs in NR (Figs. 1e, S2e). Additionally, B cell signaling to other B cells, CD4 + T cells, and CD8 + T cells was increased in R pre-ICI (Fig. 1e). These results show that the R interactome generally has more diverse communication, especially involving B and T cells. The NR interactome contains more myeloid signaling, as both a sending and receiving cell type.

### Inferred Protein-Protein Interactions Separate Responders from Non-responders, Pre-treatment and are Linked with Intracellular Pathways

**Fig. 2:**
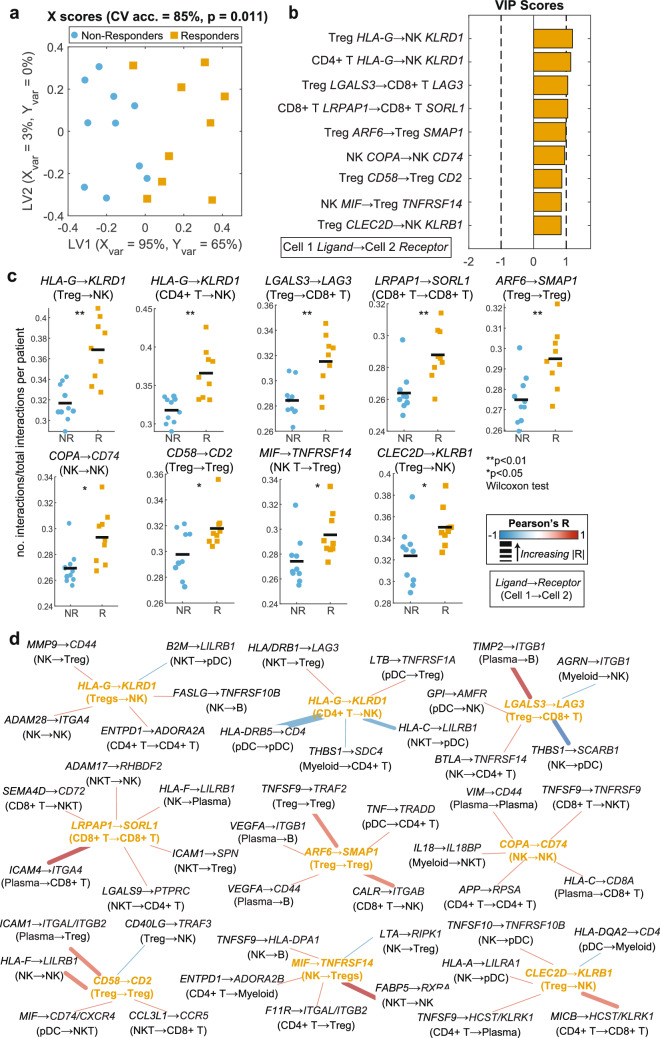
Supervised machine learning identifies ICI responder and non-responder differences in inferred interactomes pre-treatment. **a** X scores plot from the LASSO-OPLSDA model discriminating between pre-treatment TME interactomes of responders (R, orange squares) and non-responders (NR, blue circles) based on a subset of nine LASSO-selected interaction features. Each point represents one patient sample projected onto latent variables 1 and 2 (LV1 & LV2). A 5-fold cross-validation framework yielded a model accuracy score of 85%. Significance was determined by a permutation test (p = 0.011). **b** Variable importance in projection (VIP) scores are shown artificially oriented in the direction of feature loadings on LV1. |VIP|>1 indicates variables with greater than average influence on the separation between groups. Features are labeled as: Cell type A *Ligand* → Cell type B *Receptor*. **2c:** Univariate comparisons of interaction scores, defined as 1/ln(mean interaction rank), between LASSO-selected features using a Wilcoxon rank sum test. The natural log ensures that larger numeric values correspond to stronger interactions. The black line depicts the mean. **d** LASSO feature correlation network depicts the top 5 correlated features based on Pearson correlation. Center nodes are LASSO-selected features colored based on their enrichment in either responders or non-responders. Edges are weighted by the correlation coefficient such that thicker edges have stronger correlations and colored according to the correlation direction (red = positive, blue = negative)

To further characterize the specific interactions that distinguish the interaction network of R and NR pre-ICI treatment, we performed feature reduction using LASSO followed by OPLSDA on LIANA-inferred mean protein-protein and LR interaction ranks (see Methods). Mean ranks were defined as the rank of a specific interaction out of the 4701 possible interactions averaged across the five interaction inference methods that were implemented within LIANA. These mean ranks were used as input into a LASSO–OPLSDA—co-correlates network construction pipeline. Interactions with VIP scores > 1 indicate those with more than average contribution to the R/NR separation. We built another OPLSDA model with only these top VIP scored interactions and confirmed that those features together separated R from NR in pre-treatment patients. This model exhibited a CV accuracy of 85% and performed better than 989 out of 1000 models built based on randomly permuted labels (Fig. 2a).

The top interactions separating R and NR pre-treatment were enriched in R and primarily promoted an immunosuppressive tumor microenvironment, largely through Treg-mediated mechanisms (Fig. 2b, c). Including their five highest co-correlates, these interactions formed a network of 54 features (Fig. 2d) grouped into six functional categories: chemotaxis, adhesion, MHC signaling, immune activation, immune suppression, and apoptotic signaling. Detailed functions and literature justification for these interaction categories can be found in Supplemental Data 3.

Immunosuppressive interactions were the most frequent (22/54), encompassing most LASSO-selected features. Many involved known inhibitory checkpoints such as Treg *LGALS3* → CD8 + T *LAG3*, which increases cytotoxic T cell exhaustion [67–71]; Treg *CD58* → Treg *CD2*, providing costimulatory signals to enhance suppression [72, 73]; and Treg *CLEC2D* → NK *KLRB1*, an NK cytotoxicity inhibitor [74–76]. Correlated features similarly dampened NK and CD8 + T cell activity or indirectly promoted Treg proliferation, suggesting distinct mechanisms of immunosuppression in R (Treg-mediated) versus NR (myeloid and pDC-driven). Adhesion interactions were the second most common category, involving multiple B, T, and NK subsets in R. Four stabilized immune synapses between Tregs and other cell types, potentially amplifying suppression (e.g., NK T *ICAM1* → Treg *SPN*, CD4 + T *F11R* → Treg *ITGAL/ITGB2*) [77–85].

Additional interactions reflected MHC-II signaling, chemotaxis, and immune activation. For example, HLA-DR molecules, which form MHC-II, engaged CD4 more frequently in R, though atypically between pDC and myeloid cells. Chemotactic interactions promote attraction and activation of cytotoxic cell subsets, likely promoting anti-tumor activity in the TME of R (pDC *GPI* → NK *AMFR*, pDC *MIF* → NK T *CD74/CXCR4*, and NK T *CCL3L1* → CD8 + T *CCR5*) [86–88]. Finally, three immune activating interactions were enriched in R; none were enriched in NR. Two stimulating NK T cells (CD8 + T *TNFSF9* → NK T *TNFRSF9*; NK T *FABP5* → NK T *RXRA*) and one activating CD8 + T cells (CD4 + T *MICB* → CD8 + T *HCST/KLRK1*) [89–91].

**Fig. 3:**
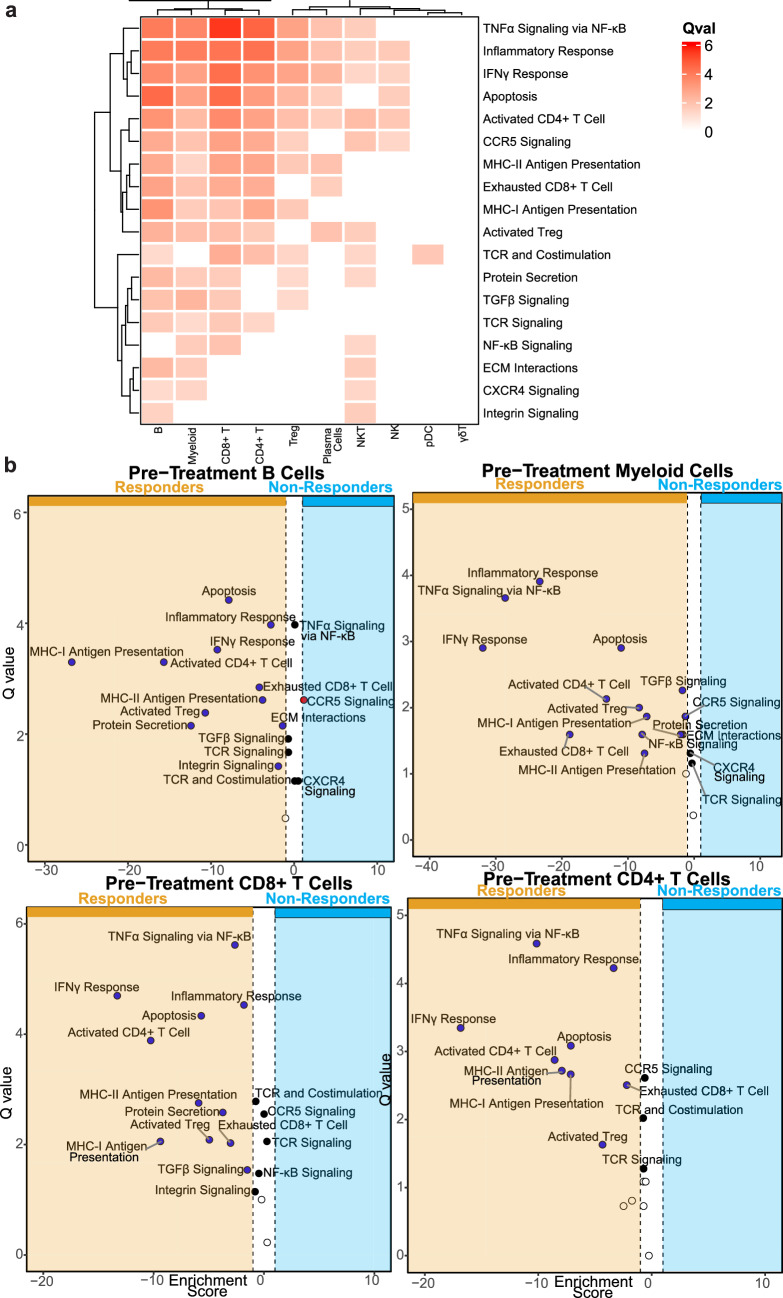
Signaling pathways up- and down-stream of inferred interactions were enriched in responders pre-treatment. **a** Heatmap of Q values comparing responder and non-responder cell-specific pathway activities in pre-ICI samples. Higher Q values indicate a more significant difference between responder and non-responder cells. **b** Volcano plot depicting Q values and fold change for pathway analysis comparing B cells, myeloid cells, CD8 + T cells, and CD4 + T cells from responder and non-responder pre-therapy samples. Filled circles identify pathways with Q values > 1, an indication that the variation of the pathway within the dataset is significant. Blue circles indicate pathways that are significantly enriched in the responder group. Black circles are pathways with significant variation, but with a fold change magnitude < 1, thus not considered enriched in either group. Red circles are pathways enriched in the non-responder group

To investigate whether cell behaviors associated with these intercellular interactions were modified in interacting cells, we performed pathway analysis to compare the transcriptional activity of signaling pathways up- or downstream of associated ligands and receptors. As such, we utilized a set of relevant MSigDB pathways (Methods, Table S5) and compared them between pre-treatment R and pre-treatment NR for each cell type identified in Fig. 1a. We observed significant differences in pathways associated with T cell function, including IFNγ response, antigen presentation via MHC-I and MHC-II, and TCR signaling (Fig. 3a). The pathways in question were strongly enriched in the R group of multiple cell types, particularly B cells, myeloid cells, CD4 + T cells, and CD8 + T cells (Fig. 3b). This shows a general upregulation in R of both inflammatory/activating pathways and cell death/exhaustion pathways that were functionally linked to the interactions enriched in R compared to NR.

### Validating the Aggregate Interaction List as a Predictor of Survival in a Separate Cohort of ICI-Treated Melanoma Patients

**Fig. 4:**
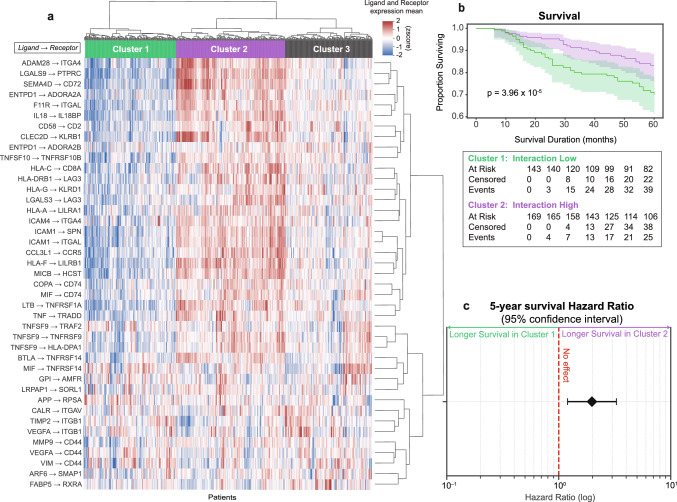
Average of significant ligand and receptor expression from machine learning models expressed in bulk RNA-seq data predicts patient survival in a separate cohort. **a** Heatmap of hierarchically clustered ligand-receptor expression scores for each patient. Columns represent individual patients and rows correspond to the average of z-scored ligand-receptor expression from bulk RNA-seq samples. Patients are classified into three groups based on expression profiles. **b** Kaplan-Meier Analysis describing overall survival for patients in Cluster 1 (Green) and Cluster 2 (Purple). The p-value was determined using the log-rank test. **c** 5 year hazard ratio describing the decreased chance of mortality for patients in Cluster 2 relative to those in Cluster 1. The red line denotes a hazard ratio of one, which indicates no effect

After identifying a compilation of interactions that differentiated R pre-treatment, we examined the survival association of these interaction pairs in a separate set of 411 melanoma patients treated with ICI, for which bulk RNA-sequencing data were available on CBioPortal [52]. The average z-score was calculated for each of the protein-protein interaction pairs that were identified by OPLSDA as top discriminants of R and NR (Fig. 2, Supplemental Data 4). Clustering based on the averages of the LR Expression pairs resulted in three distinct clusters of patients (Fig. 4a). Cluster 1, which expresses low levels of the genes involved in interactions predictive of response, has significantly worse survival than cluster 2, which expresses high levels of these genes (p < 0.01, Fig. 4b). A third cluster with a mixed phenotype was also observed, which displayed similar survival trends as cluster 1 (Fig. S3a, b). The 5 year hazard ratio analysis indicated that patients in Cluster 2 have a significantly lower risk of mortality within 5 years as compared to patients in Cluster 1, with a hazard ratio of 1.98 (95% CI 1.20–3.27), suggesting a 98% reduced risk (Fig. 4c).

### Multivariate Combinations of Inferred Interactions Separate Pre- and Post-treatment Patients

To further investigate potential interactome-based mechanisms for response and resistance, we next identified pre- to post-treatment changes in R and NR. Upon examining shifts in R and NR separately, there were no significant changes in cell type proportion pre- to post-treatment (Fig. S4a). Similarly, there were not significant differences in the number of unique interactions inferred for any of the cell type pairs pre- to post-treatment (Fig. S4b). Therefore, cell type abundances were not significant contributors to variations in inferred interactions pre- and post-ICI.

**Fig. 5:**
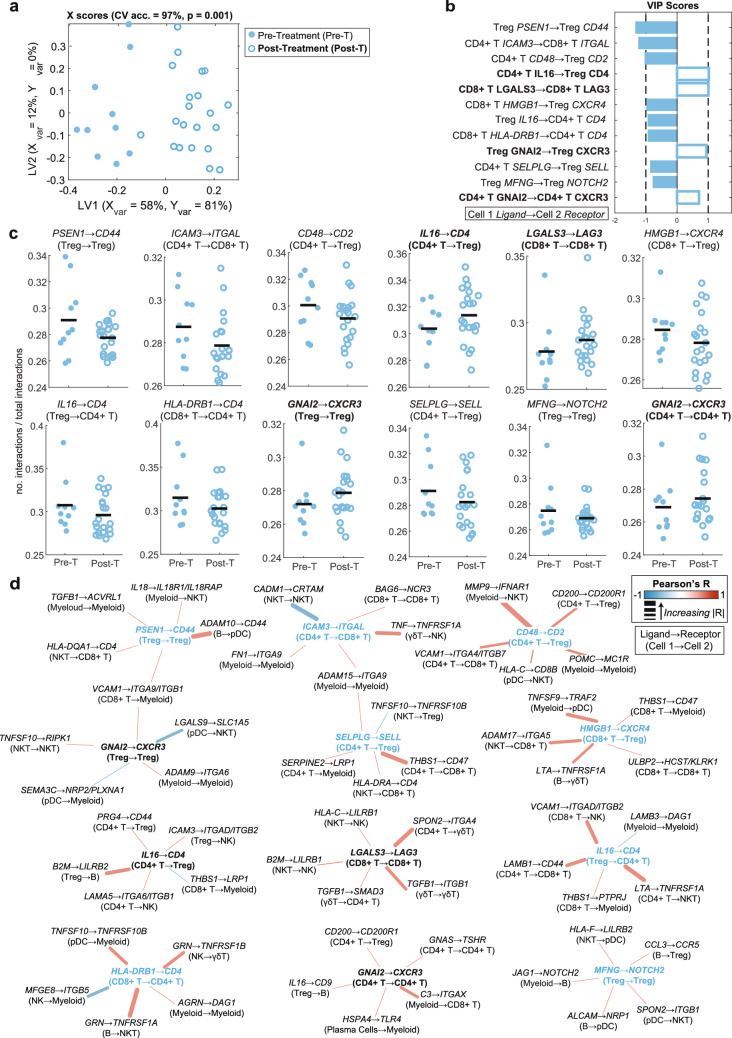
Inferred interactome changes pre- to post-treatment in non-responders. **a** X scores plot from the LASSO-OPLSDA model discriminating between the TME interactomes of non-responders pre-treatment (pre-T, filled circles) and post-treatment (post-T, open circles) based on a subset of 15 LASSO-selected interaction features. Each point represents one patient sample projected onto latent variables 1 and 2 (LV1 and LV2). A 5-fold cross-validation framework yielded a model accuracy score of 97%. Significance was determined by a permutation test (p = 0.001). **b** Variable importance in projection (VIP) scores are shown artificially oriented in the direction of feature loadings on LV1. |VIP|>1 indicates variables with greater than average influence on the separation between groups. Features are labeled as: Cell type A *Ligand* → Cell type B *Receptor*. **c** Univariate comparisons of interaction scores between LASSO-selected features using a Wilcoxon rank sum test. The black line depicts the mean. **d** LASSO feature correlation network depicts the top 5 correlated features based on Pearson correlation. Center nodes are LASSO-selected features. Blue center nodes are enriched pre-treatment, and black center nodes are enriched post-treatment. Edges are weighted by the correlation coefficient such that thicker edges have stronger correlations and colored according to the correlation direction (red = positive, blue = negative)

The ability of specific interactions to separate pre- and post-treatment patients was investigated using LASSO-OPLSDA as described previously. Despite no single interaction being significantly different pre- to post-treatment in non-responsive patients, the OPLSDA model obtained 97% CV accuracy and performed better than all 1000 randomly permuted models (Fig. 5a–c). As in the pre-ICI comparison, immune suppression and adhesion interactions dominated the correlation network, followed by less frequent chemotactic signals (Fig. 5d). Most interaction categories were more abundant in pre-ICI NR, particularly immune activation and MHC-related interactions.

Many adhesion interactions involved integrins with secondary immunomodulatory roles. One LASSO-selected feature enriched pre-ICI, CD4 + T *ICAM3 → *CD8 + T *ITGAL* (ICAM3 → LFA-1; IT*GAL* is the alpha chain) mediates early immune responses by stabilizing T cell contacts [92, 93]. Other pre-ICI correlates included myeloid-myeloid, B cell-pDC, and T-NK cell adhesion interactions. Four interactions signaled through ITGA9 in myeloid cells, which can activate FAK, MAPK, and NFκB pathways [94–96], promoting a pro-tumor macrophage phenotype that was downregulated post-treatment. Post-ICI adhesion was dominated with myeloid interactions likely contributing to immunosuppressive phenotypes upregulated after therapy in NR [97–100].

Immune suppression mechanisms encompassed checkpoint interactions, Treg-mediated suppression, and innate immune inhibition. Four LASSO-selected features were Treg-suppressive: CD4 + T *SELPLG* → Treg *SELL* [101], Treg *MFNG* → Treg *NOTCH2* [102], and Treg *PSEN1* → Treg *CD44* [83] (enriched pre-ICI) and CD8 + T *LGALS3* → CD8 + T *LAG3* [69] (enriched post-ICI). Correlated pre-ICI suppressive interactions were mainly innate immune-mediated with only two Treg-mediated interactions (CD4 + T *PRG4* → Treg *CD44* [103] and Treg *B2M* → B *LILRB2* [104]). Post-ICI correlates included three innate immune inhibition and four T cell suppression interactions.

Chemotactic interactions accounted for five of 11 LASSO-selected features, most promoting Treg recruitment: CD4 + T *IL16* → Treg *CD4* [105], CD8 + T *HMGB1* → Treg *CXCR4* [106], and B *CCL3* → Treg *CCR5* [107]. Post-ICI samples instead showed enrichment for non-Treg chemotaxis. Pre-ICI samples also included single myeloid-attracting (CD4 + T *SERPINE2* → myeloid *LRP1* [108]) and one CD4 + T cell chemoattraction (Treg *IL16* → CD4 + T *CD4* [105]), but were otherwise involved in Treg-directed chemotaxis.

**Fig. 6:**
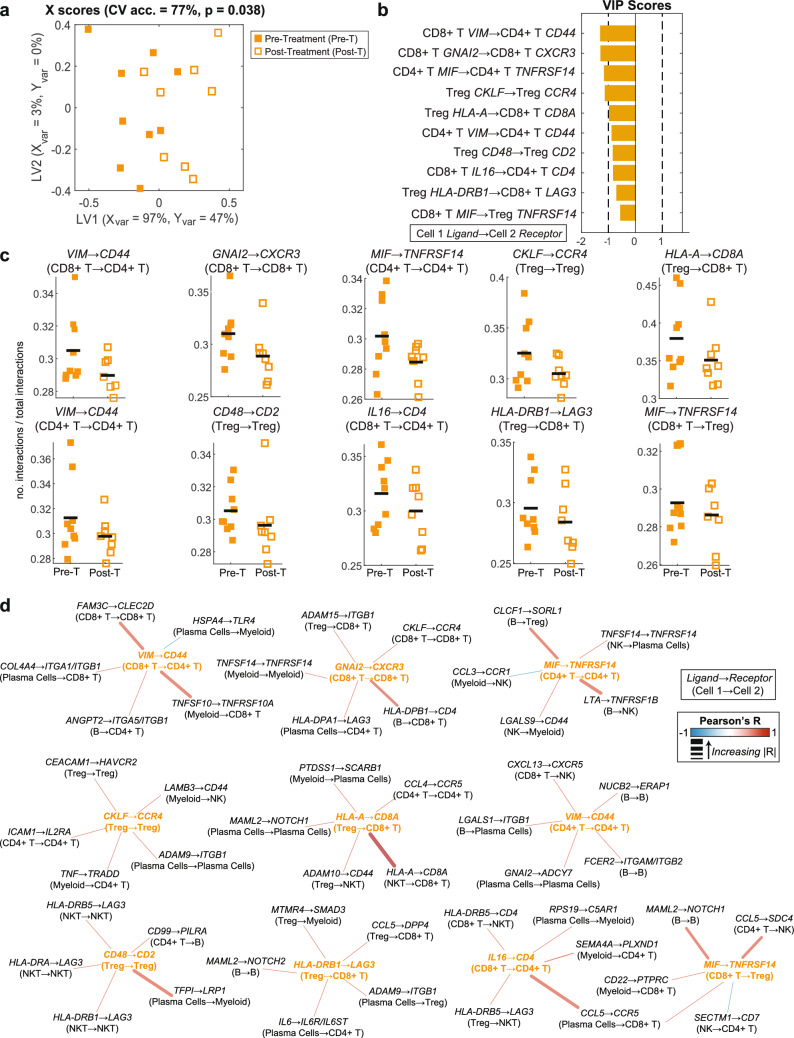
Inferred interactome changes pre- to post-treatment in responders. **a** X scores plot from the LASSO-OPLSDA model discriminating between the TME interactomes of responders pre-treatment (pre-T, filled squares) and post-treatment (post-T, open squares) based on a subset of ten LASSO-selected interaction features. Each point represents one patient sample projected onto latent variables 1 and 2 (LV1 & LV2). A fivefold cross-validation framework yielded a model accuracy score of 77%. Significance was determined by a permutation test (p = 0.038). **b** Variable importance in projection (VIP) scores are shown artificially oriented in the direction of feature loadings on LV1. |VIP|>1 indicates variables with greater than average influence on the separation between groups. Features are labeled as: Cell type A *Ligand* → Cell type B *Receptor*. **c** Univariate comparisons of interaction scores between LASSO-selected features using a Wilcoxon rank sum test. The black line depicts the mean. **d** LASSO feature correlation network depicts the top 5 correlated features based on Pearson correlation. Center nodes are LASSO-selected features. Orange center nodes are enriched pre-treatment, and black center nodes are enriched post-treatment. Edges are weighted by the correlation coefficient such that thicker edges have stronger correlations and colored according to the correlation direction (red = positive, blue = negative)

Similarly, in responding patients multivariate OPLSDA successfully separated pre- and post-treatment groups with 77% CV accuracy though no individual interaction was significantly different (Fig. 6a, c). Of the selected interactions, MHC-II—LAG3, Treg activation, and CD8 T cell recruitment are predictive of pre-treatment status (Fig 6b). The correlated interaction network revealed enrichment of immunosuppressive, adhesion, and chemotactic interactions pre-treatment, similar to patterns observed in NR pre vs. post (Fig 6d). Suppressive interactions frequently involved CD8 + T cell inhibition and Treg activation such as Treg *HLA-DRB1* → CD8 + T *LAG3* [109, 110] and Treg *CD48* → Treg *CD2* [73, 111]). Correlated features included multiple NK T cell *HLA-DR* → *LAG3* interactions and several plasma cell-mediated suppression (*e.g.* plasma cell *RPS19* → myeloid *C5AR1* [112]).

Adhesion interactions in this comparison prominently signaled through CD44 in various cell types (CD4 + T/CD8 + T *VIM* → CD4 + T *CD44* [113, 114], NK *LGALS9* → myeloid *CD44* [115, 116], Treg *ADAM10* → NK T *CD44* [117, 118], myeloid *LAMB3* → NK *CD44* [119]). CD44 provides adhesion to the extracellular matrix and neighboring cells while also delivering activation and survival cues, especially to T cells [119]. ITGB1 was a common ligand for the remaining adhesion interactions, mediating interactions between CD8 + T cells, CD4 + T cells, Tregs, and plasma cells, and enhancing T cell activation and effector function [120–122].

Chemotactic interactions were predominantly elevated pre-ICI, except for myeloid *CCL3* → NK *CCR1*, which increased post-ICI. Seven of ten pre-ICI chemotactic interactions promoted CD4 + or CD8 + T cell recruitment, while the others recruited NK cells (two interactions) or Tregs (one interaction). Collectively, these findings suggest that prior to therapy the immune environment is highly mobile, but chemotactic signaling declines following treatment.

### Aspects of Interactome Shifts with Treatment are Conserved in Additional scRNA-seq Cohort

**Fig. 7:**
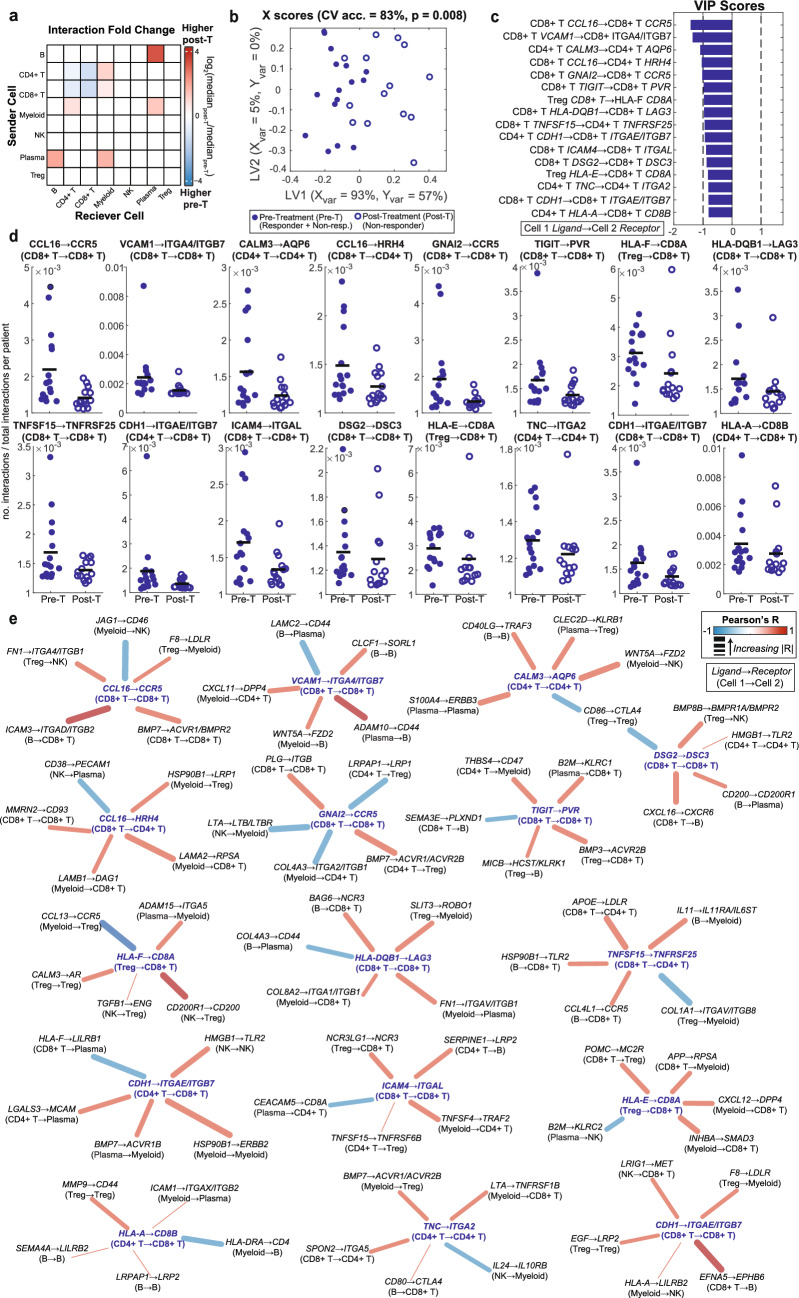
Inferred interactome differences in pre-treatment responders and non-responders compared to non-responders, post-treatment from Jerby et al. **a** Heatmap depicting the fold change of the median number of pairwise cell type interactions between pre-treatment patients (including responders and non-responders) and post-treatment patients (non-responders only), with 0.001 added to both median values to prevent division by zero or attempting to take the log of a zero value. The fold change is log2 normalized. Red indicates cells which interact more frequently post-treatment, and blue indicates cells which interact more frequently pre-treatment. **b** X scores plot from the LASSO-OPLSDA model discriminating between the TME interactomes of patients pre-treatment (pre-T, filled circles) and non-responders post-treatment (post-T, open circles) based on a subset of ten LASSO-selected interaction features. Each point represents one patient sample projected onto latent variables 1 and 2 (LV1 & LV2). A 5-fold cross-validation framework yielded a model accuracy score of 83%. Significance was determined by a permutation test (p = 0.008). **c** Variable importance in projection (VIP) scores are shown artificially oriented in the direction of feature loadings on LV1. |VIP|>1 indicates variables with greater than average influence on the separation between groups. Features are labeled as: Cell type A *Ligand* → Cell type B *Receptor*. **d** Univariate comparisons of interaction scores between LASSO-selected features using a Wilcoxon rank sum test. The black line depicts the mean. **e** LASSO feature correlation network depicts the top 5 correlated features based on Pearson correlation. Center nodes are LASSO-selected features. Dark blue center nodes are enriched pre-treatment. Edges are weighted by the correlation coefficient such that thicker edges have stronger correlations and colored according to the correlation direction (red = positive, blue = negative).

To determine the generalizability of our observations, we repeated analyses in an additional scRNA-seq dataset of melanoma patients treated with ICI [27]. This dataset contains pre-treatment patients with no response data and post-treatment NR patients. As observed in our discovery dataset, there were no significant differences in cell type proportions or interaction frequencies pre- to post-treatment (Fig. S5a, b). When compared against all pre-ICI and NR post-ICI patients from the discovery dataset, the cell type proportions were not significantly different between our discovery and validation datasets (Fig. S5a). However, interaction frequencies varied, with CD8 + T cell-involving pairs differing more across the two datasets at matched timepoints (Fig. S5b). In the validation dataset, post-ICI samples showed elevated proportions of unique Treg and B cell interactions, while CD4 + T and CD8 + T interactions decreased (Fig. 7a).

Inferred interactions were able to separate pre- and post-treatment patients with 83% accuracy, despite the likely mixed response of the pre-treatment group in this dataset (Fig. 7b). As in the R and NR pre *vs.* post comparisons, most interactions were enriched pre-ICI in the validation cohort (Fig. 7c–e). The most common category of interactions was those with immunosuppressive functions. This includes two LASSO-selected features that involve the checkpoint molecules *TIGIT* and *LAG3* (CD8 + T *TIGIT* → CD8 + T *PVR* [123, 124], CD8 + T *HLA-DQB1* → CD8 + T *LAG3* [70, 125]). Both of these interactions, in addition to the correlated interaction B *CD80* → CD8 + T *CTLA4*, point to classical checkpoint-mediated inhibition of CD8 + T cell activity as enriched pre-ICI [123, 125, 126]. Additional suppressive interactions targeted Treg and myeloid cell activity via a variety of mechanisms. Overall, the mixed response pre-ICI group exhibited more suppressive interactions than the post-ICI NRs, consistent with the discovery dataset findings.

Adhesion interactions were the next most prevalent, were enriched pre-treatment, and featured activating signaling through ITGB-containing complexes on CD8 + T and NK cells [127, 128]. Apart from three myeloid-activating interactions enriched post-ICI, immune activating interactions were enriched pre-ICI and included interactions activating CD4 + T cells and CD8 + T cells (ex. CD8 + T *CCL16* → CD4 + T *HRH4* [129], CD8 + T *TNFSF15* → CD4 + T *TNFRSF25* [130], myeloid *LTA* → CD8 + T *TNFRSF1B* [131]). Both the adhesion and the immune activation interactions share characteristics with pre-ICI R vs NR (Fig. 1) and NR pre- vs post-ICI (Fig. 5), in line with the presumed mixed response composition of the validation dataset pre-ICI group.

## Conclusion and Discussion

Based on our analyses of two independent scRNA-seq data sets, we identified intercellular interactions that form a predictive profile associated with response to ICI in advanced melanoma. We further examined how these interactions shift pre- to post-treatment in R and NR patients, providing insight into potential mechanisms of therapeutic response and resistance. While single interactions alone did not fully separate patient groups, network-level changes in cellular communication robustly predicted treatment outcomes. Notably, we found that the interactome difference between R and NR is more pronounced than treatment-induced remodeling within each group, suggesting that pre-existing interaction states may strongly influence therapeutic success (compare univariate plots of Figs. 5, 6 to Fig. 2).

Our analyses build upon the original reports from which these datasets were sourced, which primarily focused on cell type abundances and transcriptional phenotypes [26, 27]. Our network-level approach highlights distinct interaction patterns between immune populations. For example, we observe enriched B–T cell communication, CD8 + T cell adhesion and migration signals, and Treg-mediated immunosuppressive interactions in responders (Figs. 1d**, **2). This combination of supportive B cell signaling, migration-capable CD8 + T cells, and regulated immune suppression suggests a tumor microenvironment primed for adaptive immune cooperation. Responders also showed greater CD8 + signaling diversity compared to NR, despite similar CD8 + cell frequencies (Fig. 1b, c), reinforcing that communication diversity—rather than abundance—may underlie durable responses. This aligns with imaging studies suggesting that spatial dynamics and communication, rather than cell abundance, drive anti-tumor efficacy [132]. Prognostic relevance of these interactions was further confirmed by improved survival in bulk RNA-seq datasets (Fig. 4).

Differences in CD4 + T cell subsets also emerged as important contributors to interactome alterations. In pre-treatment samples, responders had higher fractions of naïve and Th2 CD4 + T cells, as well as stem-like populations (Fig. S2b, c), suggesting a more dysfunctional helper T cell population in NR before ICI is initiated. However, non-responders showed an increase in Th2 fractions post-treatment (Fig. S2b). Literature links elevated Th2 cytokines to poor outcomes and a shift of CD4 + T cell phenotypes from a Th2 to a Th1 response with ICI [133, 134], and our findings support this.

Pre- versus post-treatment analyses reveal that non-responders develop stronger myeloid-driven suppressive signaling and experience losses in activating NK and T cell interactions (Fig. 5). Combined with increased Th2-like CD4 + T cell fractions post-ICI (Fig. S2b), these changes point to progressive dysfunction in cytotoxic and helper T cell compartments, consistent with proposed roles of Th2 skewing and myeloid-driven suppression in ICI resistance [135, 136]. The elevated number and strength of unique myeloid interactions (Fig. 1d) further support their association with treatment failure.

Responders followed a distinct trajectory pre-treatment: their tumors showed high immune mobility but active suppression, which shifted post-treatment toward a less inhibited state (Fig. 6). Chemotactic interactions in responders involved CD8 + and NK cells, while in non-responders they were dominated by Tregs and CD4 + cells. Responders also trended toward reduced Th2-like helper T cells post-treatment (Fig. S2b, c), though this was not accompanied by increased Th1 fractions as expected. Instead, exhausted CD8 + T cells emerged, potentially reflecting initial activation followed by partial functional decline, consistent with acquired resistance in a subset of melanoma patients [137]. These results suggest that ICI therapy triggers gradual, coordinated remodeling of the tumor microenvironment’s communication network, with therapeutic outcomes shaped by baseline interactome states.

In the validation dataset [27], we observed similar functional patterns of interactions despite cohort-specific differences in interaction frequencies (Fig. 7). This discrepancy likely reflects differences in cohort composition: specifically, the comparison here was between a pre-ICI group with unknown response status and a post-ICI group composed entirely of NR (Fig. 7) rather than a direct NR pre- *vs.* post-ICI comparison (Fig. 6). For example, B cell and plasma cell interactions were elevated in R pre-ICI of the discovery dataset as compared to the mixed pre-ICI group in the validation dataset (Figs. 1d, 7a). While this prevents direct validation of treatment-induced interactome remodeling, the preservation of network motifs between datasets supports that the differences in the interactome between R and NR are more pronounced than longitudinal changes within response groups.

The current work is limited by several inherent characteristics of intercellular communication inference and the datasets available in this tumor context. First, interaction inference is based on RNA expression and may not fully capture protein-level or post-translational modifications influencing cell communication. Second, single-cell data sparsity, which propagates through interaction inference, necessitated an imputation step to enable PLSDA analysis. While imputation is valuable for mitigating dropout, it introduces uncertainty by estimating missing values that may represent true biological zeros. Furthermore, because imputation relies on patterns in the observed data to estimate missing values, it can dampen biological variability. This effect is especially pronounced when the proportion of missing values is high. We partially addressed this by thresholding interactions for completeness and validating results with permutation-based significance testing. However, the thresholding step introduces an additional limitation: only interactions with sufficient completeness across samples were analyzed, potentially excluding rare but biologically relevant interactions.

Third, our iterative manual pruning of LASSO-selected features reduces the risk of biologically implausible predictions but introduces potential confirmation bias. To minimize this, pruning criteria were predefined and biologically infeasible interactions are fully documented (Supplementary Data 3). Fourth, modest sample sizes, particularly for matched pre/post-treatment comparisons, limit generalizability and might contribute to overfitting. To mitigate this risk, we employed repeated cross-validation, permutation-based p-values, and feature stability checks. Nonetheless, larger and more balanced cohorts are needed to further validate these findings. Additionally, tumors originated from diverse anatomical sites [138, 139], which may influence immune composition and intercellular signaling. Future studies incorporating site-stratified analyses could help disentangle these effects.

Due to an absence of available independent scRNA-seq datasets with pre-ICI response metadata, pre-treatment analyses could not be directly validated in an independent cohort. However, expression of ligands and receptors associated with response were associated with improved survival in pre-treatment bulk RNA-seq of patient tumors treated with ICIs. Validation of pre- to post-treatment changes was performed using a comparison between pre-All samples (an unknown mix of R and NR) and post-NR samples. While not a one-to-one matched comparison, this analysis provided complementary evidence into immune interactome shifts that appear robust to cohort-specific variation.

Looking ahead, considering the top differentially inferred interactions as regulators of a dynamic immune cell state transitions offers a promising framework to study the evolution of a pro-tumor melanoma TME. Future work should explore upstream regulators and downstream pathways connecting these intercellular interactions to other immune evasion mechanisms. Experimental testing of candidate interactions, such as blocking ligand-receptor interactions in vitro to assess effects on CD8 + T cell cytotoxicity or CD4 + T cell cytokine production, will be critical to establish functional relevance.

Additional mechanistic insights may also come from studying post-translational modifications that influence cellular crosstalk. For example, altered glycosylation of tumor or immune secreted or cell surface proteins, including galectin-mediated signaling, may shape chemotactic and contact-dependent interactions. Importantly, spatially resolved transcriptomics or proteomics methods are needed to infer whether the interacting cells are physically co-localized within tumors. Because the intercellular communication relies on both molecular expression and spatial proximity, such experimental validation represents a critical next step.

By advancing toward a system-level view of the TME system as a dynamic communication networks, we can develop a more comprehensive model of altered cellular crosstalk before and after immune checkpoint blockade, which in turn leads to better informed prognostic predictions and rationally guide therapeutic strategies for increased immunotherapeutic efficacy.

## Supplementary Information

Below is the link to the electronic supplementary material.

Supplementary file1 (DOCX 2260 kb)

Supplementary file2 (XLSX 20 kb)

Supplementary file3 (XLSX 34 kb)

Supplementary file4 (CSV 0 kb)

Supplementary file5 (XLSX 69 kb)

## Data Availability

Datasets from Sade-Feldman et al. and Jerby-Arnon et al. were previously published and available at GEO (GSE120575; GSE115978). Codes for the LASSO feature selection OPLSDA analysis used in this work are available at https://github.com/Dolatshahi-Lab/Elastic-Net-for-PLSR-DA and https://github.com/Dolatshahi-Lab/PLSR-DA. Additional processed data are available as Supplemental Data and from the authors upon request.
