# Supplementary material for: Uncovering Cellular Interactome Drivers of Immune Checkpoint Inhibitor Response in Advanced Melanoma Patients
Source: Cell Mol Bioeng. 2025 Sep 8;18(5):519–41. doi: 10.1007/s12195-025-00857-y (PMC12579638; doi:10.1007/s12195-025-00857-y)
Supplement: Supplementary file 1 — Supplementary file1 (DOCX 2260 kb) [file 12195_2025_857_MOESM1_ESM.docx]

**Supplementary Information**

**Table S1: Gene expression markers used in cell type identification**

| **Cell Type** | **Markers** |
| --- | --- |
| CD8 T cells^1^ | CD8A^2,3^, GZMB^2^, TRAC^4^, CD3E^2,3,5,6^ |
| CD4 T cells^7^ | CD4^2,3^, CCR4^8^, CD28^9^, IL13^10^, (- FOXP3^11^) |
| B cells^12^ | PXK^13^, HLA-DRA^14^, MS4A1^2,3^, CD19^2,3^ |
| Regulatory T cells (Tregs)^15^ | FOXP3^11^, IKZF2^16^, IL2RA^2,3^, TNFRSF4^17^ |
| NK cells^18^ | NCAM1^2,3^, FCGR3A^3,19^, GNLY^20^, KLRD1^2^, (- CD3D/E^21^) |
| NK T cells^22^ | CD3E^23^, CD8A^23^, KLRF1^24^, FCG3A^25^, NKG7^24^, KLRD1^24^ |
| γδ T cells^22^ | CD3E^26^, GZMH^26^, TRDC^27^, TRGC1^27^, TRGC2^27^, (-CD4^24^, -CD8A^24^) |
| Myeloid^28,29^ | CD14^2,3^, CD68^2^, CD163^2,3^, LYZ^30^ |
| Plasma cells^31^ | CD38^32^, CD79A^33^, (- CD19^34,35^, - CD20^36,37^) |
| Plasmacytoid Dendritic Cells (pDCs)^38^ | IL3RA^2,3^, CLEC4C^2,3^, LILRA4^3,39^, PLD4^40,41^ |

Citations in the left Cell Type column refer to resources that provided general cell typing guidance and were used for additional marker genes. Citations in the right Markers column represent papers identifying the specific genes listed there as markers of a particular cell type.

**Table S2: Gene expression markers used in T cell clustering and myeloid cell clustering**

| **Cell Type** | **Markers** |
| --- | --- |
| T cell markers^42^ | |
| Treg ^43^ | CD4, FOXP3, IL2RA |
| CD8_Exh ^44,45^ | PRF1, GZMB, TOX, PDCD1, LAG3, TIGIT, HAVCR2 |
| CD8_pEx ^44,45^ | PRF1, GZMB, TCF7, TOX, PDCD1, LAG3, TIGIT, HAVCR2 |
| CD8_Eff ^46,47^ | NKG7, GNLY, CCL5 |
| γδ T ^48–50^ | TRDC, TRGC1 |
| CD4_Naive ^51,52^ | CD4, TCF7, LEF1, CCR7, SELL |
| Stemlike ^53,54^ | CCR7, TCF7, FOXP1, (-TIGIT, -PDCD1, -LAG3, -TOX) |
| MAIT ^48–50^ | KLRG1, TRAV1, TRAV2 |
| Th1 ^55–57^ | CD4, RUNX3, ANXA3, IFNG |
| Th2 ^55–57^ | CD4, LIMA1, IL4R, GATA3 |
| Myeloid cell markers^58–60^ | |
| C1QC^61^ | C1QB, C1QC, CD206, HLA-DR, SEPP1, APOE, FOLR2, (-SPP1) |
| CLEC12^62^ | CLEC12A, CCR2, LY6C2, S100A6 |
| AQP9^63^ | AQP9, FN1, SPP1, NDRG1 |

Citations listed for T cell markers and Myeloid cell markers were used to guide all subtypes.

**Table S3: Choice of Imputation Method, NRMSE on pre-ICI discovery mean rank matrix, 70% interaction threshold**

| **Imputation Method** | **Transposed Mean Rank Matrix** | **NRMSE** |
| --- | --- | --- |
| Random Forest | Yes | 0.341 |
| Random Forest | No | 0.529 |
| MICE PMM | Yes | 0.509 |
| MICE PMM | No | NA – did not converge, too few samples to provide plausible donor values |
| kNN | Yes | 0.455 |
| kNN | No | NA – error, no samples with complete data, can’t compute nearest neighbors |

**Table S4: Choice of Interaction Threshold, NRMSE and Retained Interactions of Imputation**

| **Comparison** | **Minimum Proportion of Samples with Observed Interaction Scores** | **Retained Interactions** | **Percent Zeros** | **NRMSE** |
| --- | --- | --- | --- | --- |
| Pre R *vs.* NR | 0.7 | 1037 | 13.85 | 0.341 |
| NR Pre *vs.* Post | 0.92 | 476 | 3.28 | 0.290 |
| R Pre *vs.* Post | 0.9 | 285 | 4.83 | 0.253 |
| Validation Pre *vs.* Post | 0.72 | 1294 | 15.31 | 0.335 |

**Table S5: Selected Pathways**

| Interaction Category | mSigDB Pathways |
| --- | --- |
| Immune Suppression | HALLMARK_TGF_BETA_SIGNALING  BIOCARTA_NFKB_PATHWAY  GSE9650_EFFECTOR_VS_EXHAUSTED_CD8_TCELL_DN  GSE15659_RESTING_VS_ACTIVATED_TREG_DN |
| Apoptotic | HALLMARK_APOPTOSIS  HALLMARK_TNFA_SIGNALING_VIA_NFKB |
| MHC | REACTOME_CLASS_I_MEDIATED_ANTIGEN_PROCESSING_PRESENTATION  REACTOME_MHC_CLASS_II_ANTIGEN_PRESENTATION |
| Other | HALLMARK_PROTEIN_SECRETION |
| Adhesion | KEGG_ECM_RECEPTOR_INTERACTION  BIOCARTA_INTEGRIN_PATHWAY |
| Chemotaxis | BIOCARTA_CXCR4_PATHWAY  BIOCARTA_CCR5_PATHWAY |
| Immune Activation | HALLMARK_INFLAMMATORY_RESPONSE  HALLMARK_INTERFERON_GAMMA_RESPONSE  WP_T_CELL_RECEPTOR_AND_COSTIMULATORY_SIGNALING  BIOCARTA_TCR_PATHWAY  GSE13738_RESTING_VS_TCR_ACTIVATED_CD4_TCELL_DN |
| Note: Pathways were selected based on the interactions that were identified in the pre-ICI R *vs.* NR comparison and their corresponding functional categories. These pathways may have other biological functions depending on the circumstances. | |

**Figure S1: Additional scRNAseq quality control and cell typing information**

**a.**


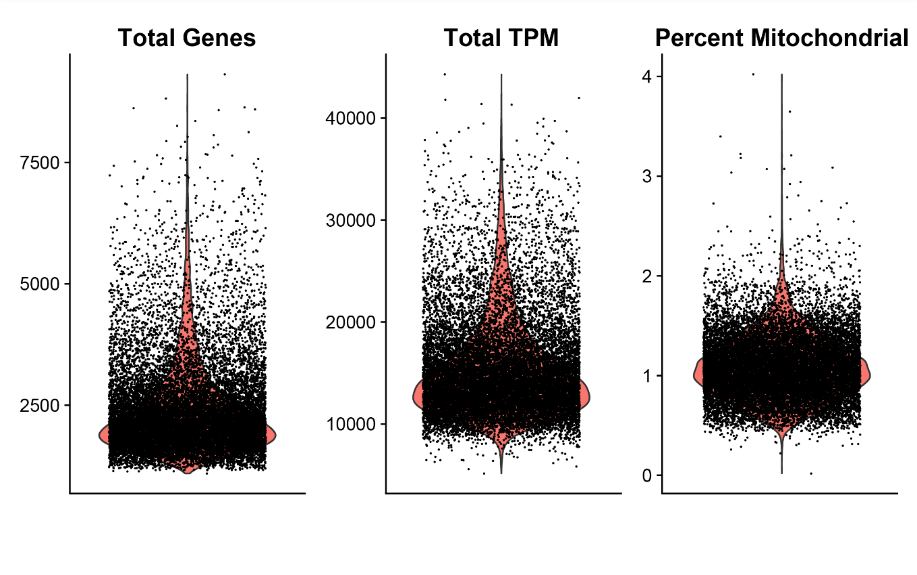


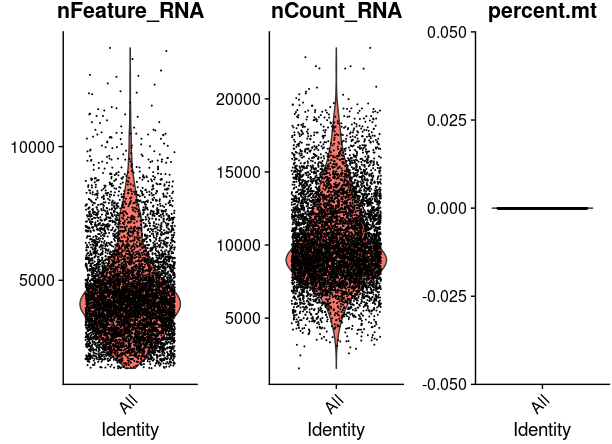
**b**.

**c.**

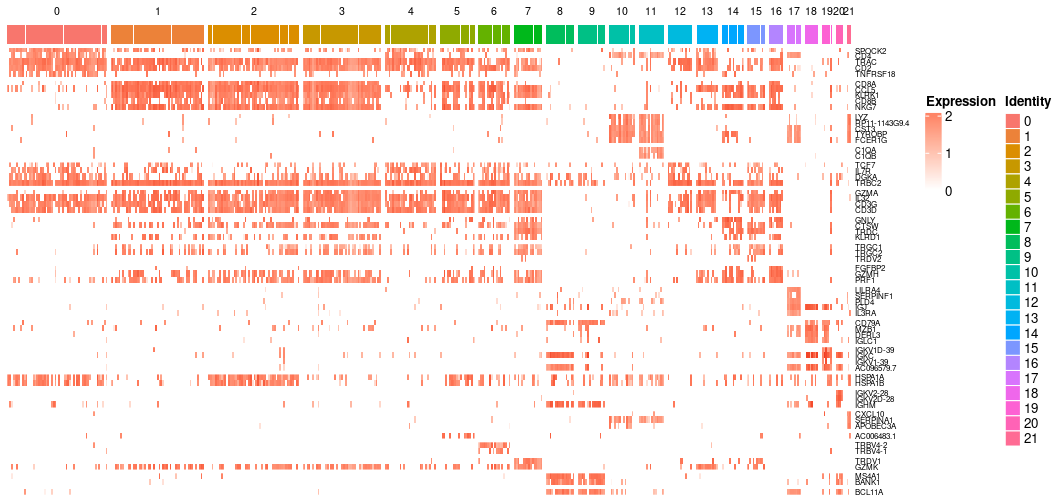


d.
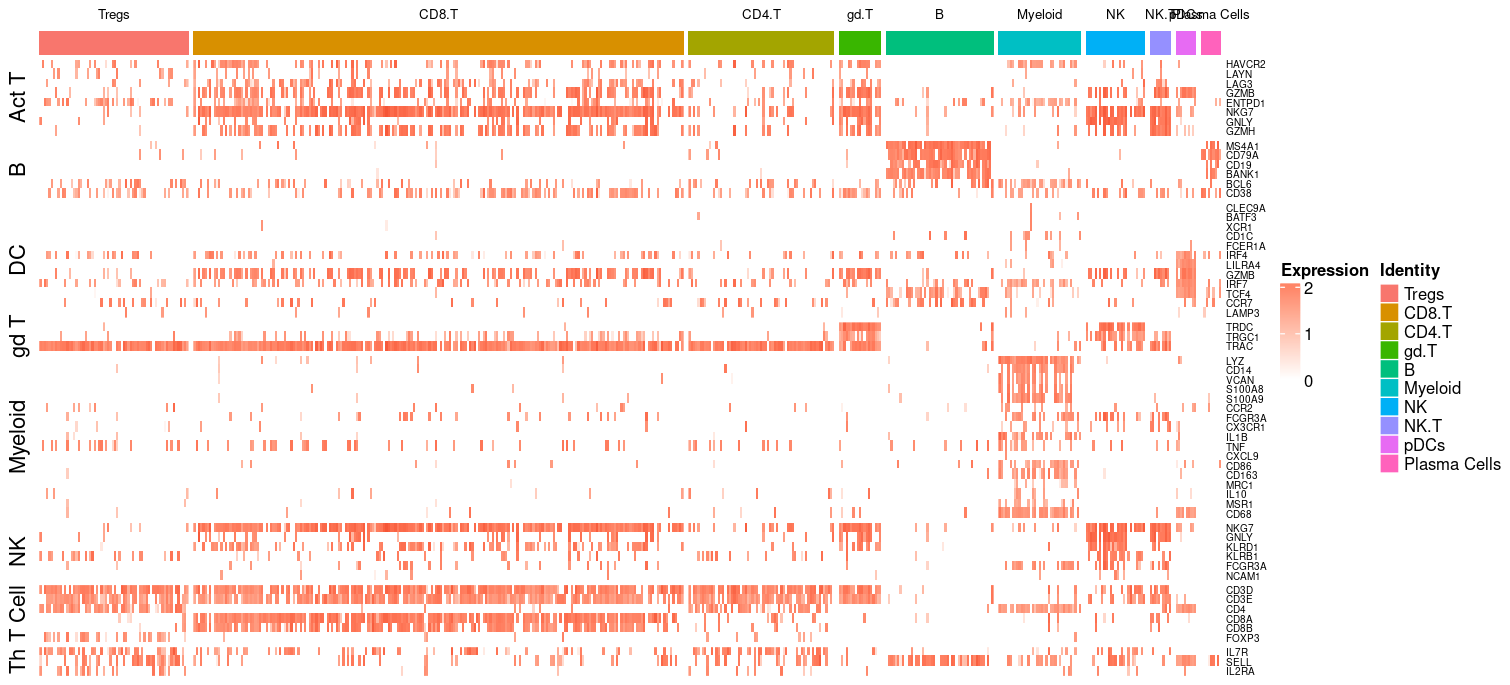


e.


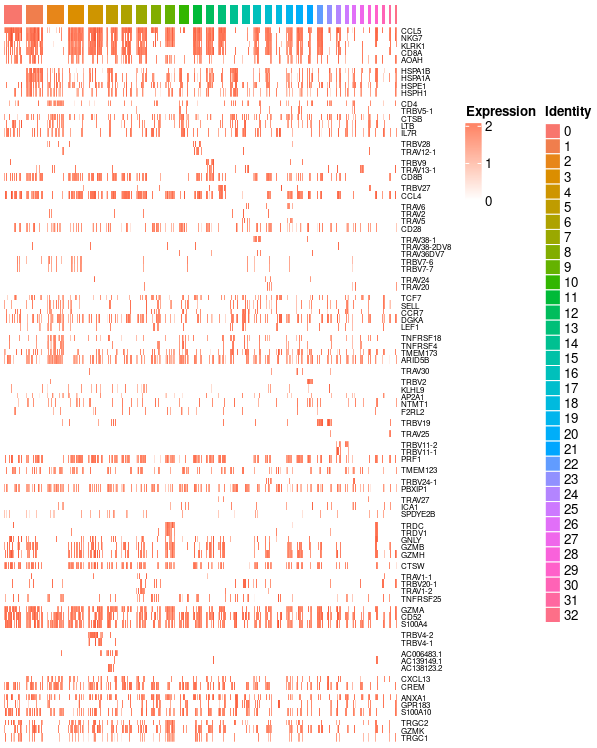


f.


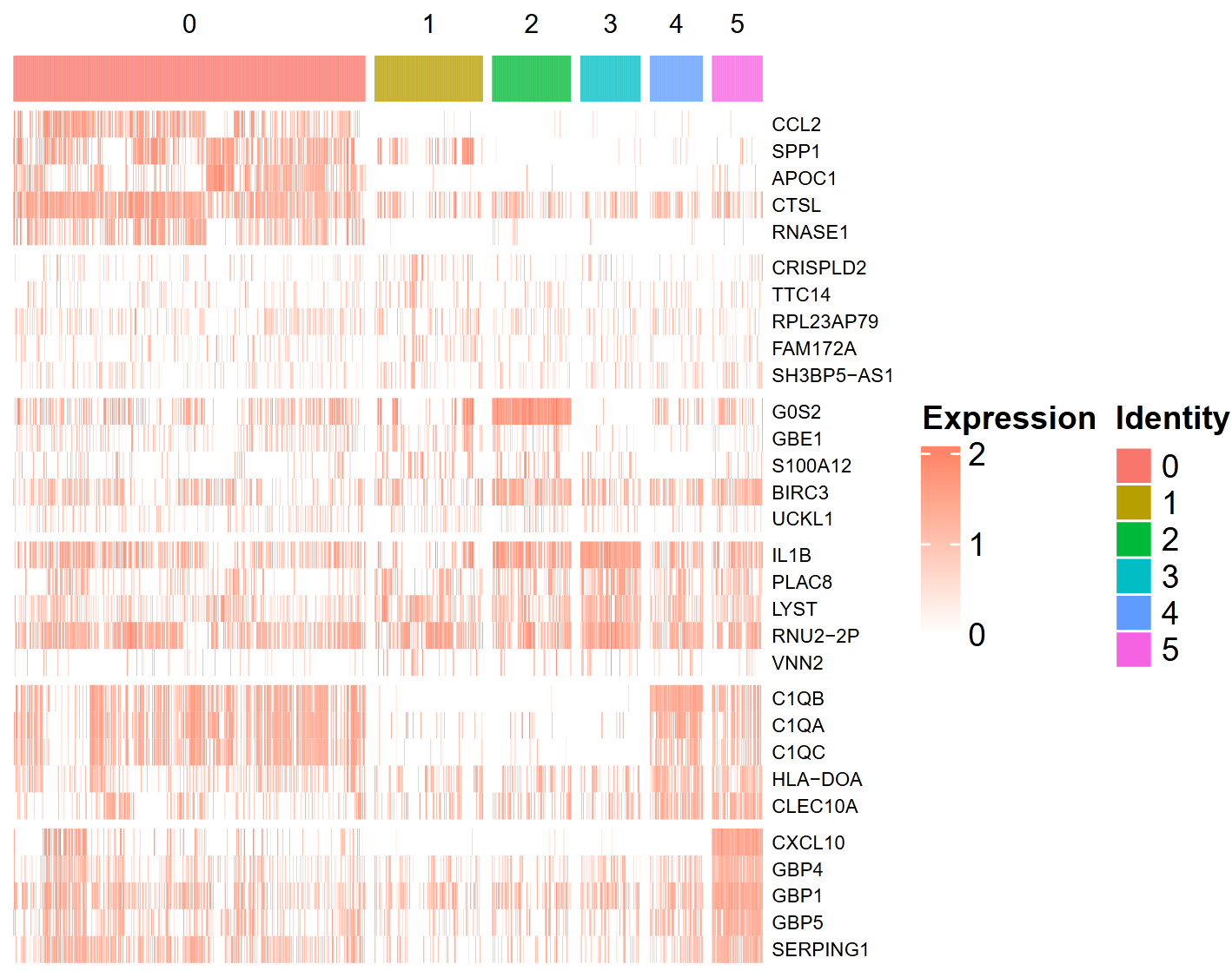


**a.** Quality control plots for Sade-Feldman dataset. Cells with Total Genes (nFeature_RNA) > 500, Total Genes < 3000, and Percent Mitochondrial Genes <2 were retained for downstream analysis.

**b.** Quality control plots for Jerby-Arnon dataset. Dataset obtained from GEO had zero mitochondrial genes included. Cells with nFeature_RNA > 500 and nFeature_RNA < 7500 were retained for downstream analysis.

**c.** Differentially expressed genes for each cluster used to supplement expression of canonical cell type markers in cell type identification process.

**d.** Expression of canonical cell type markers in the defined cell type clusters.

**e.** Expression of markers used to determine T cell subsets

**f.** Expression of markers used to determine myeloid subsets

**Figure S2: Univariate comparison of cell subsets and interaction frequencies**

**a.**


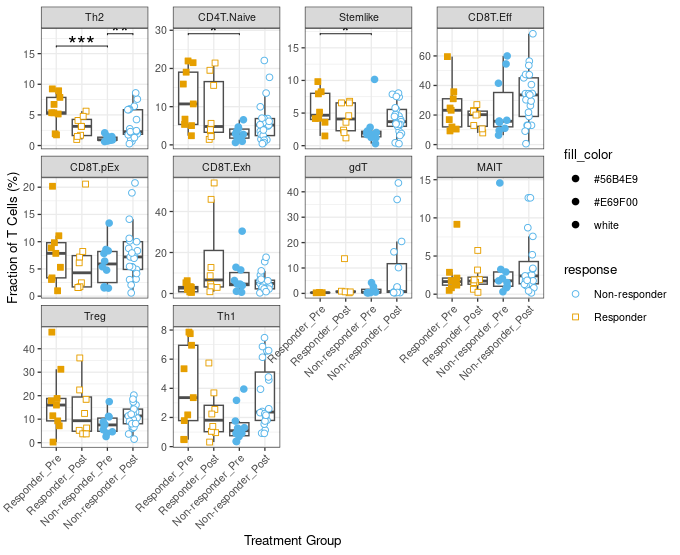

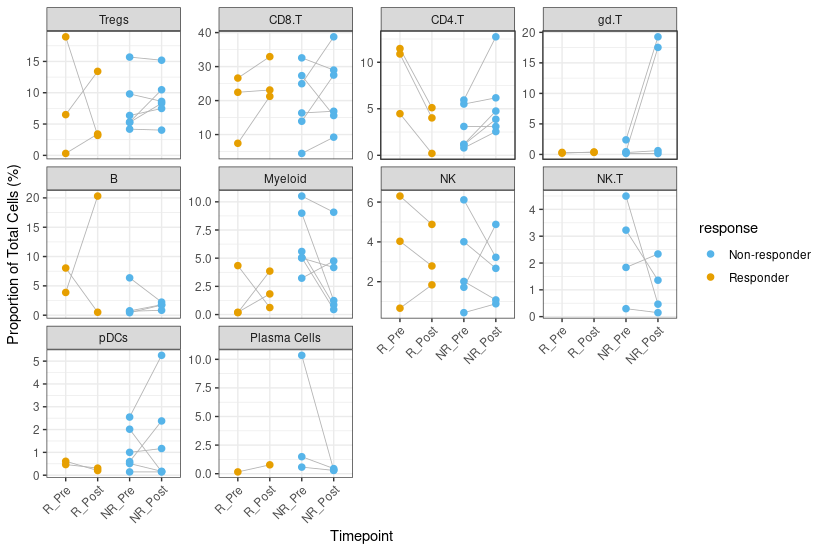


**b.**

**c.

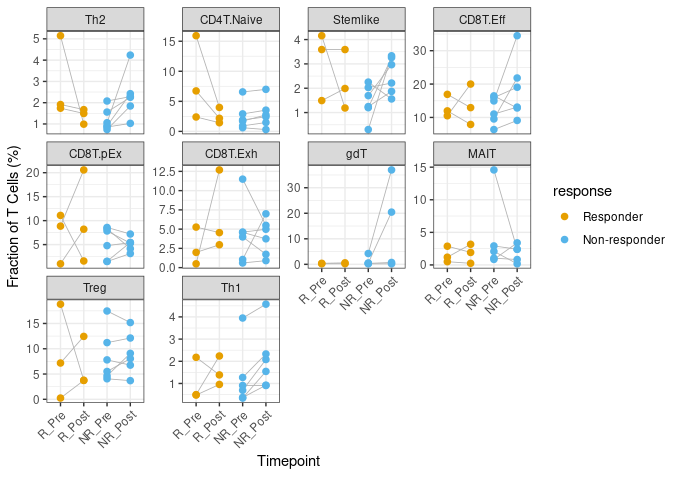
**

**d.**


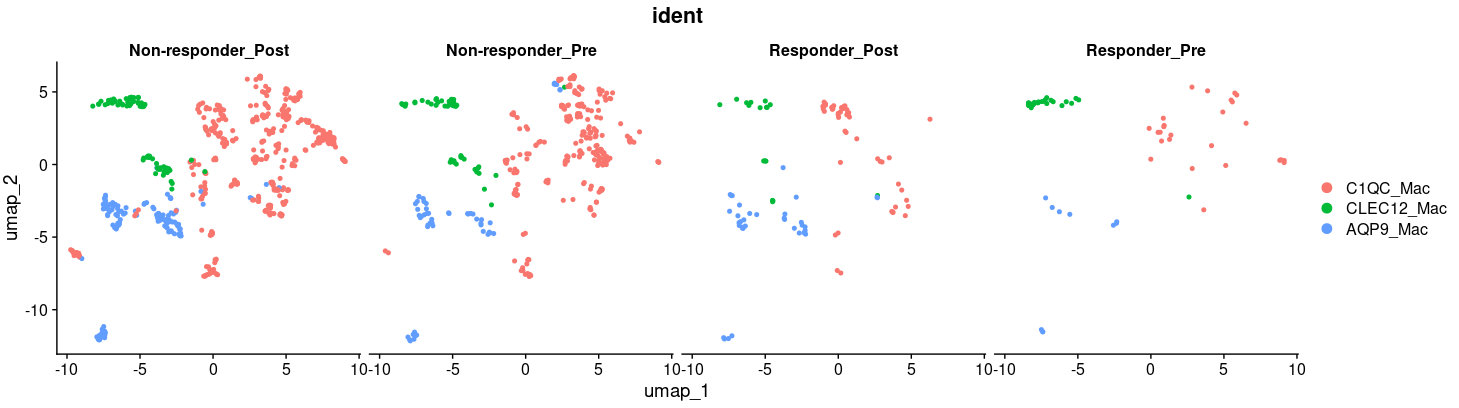


**e.**
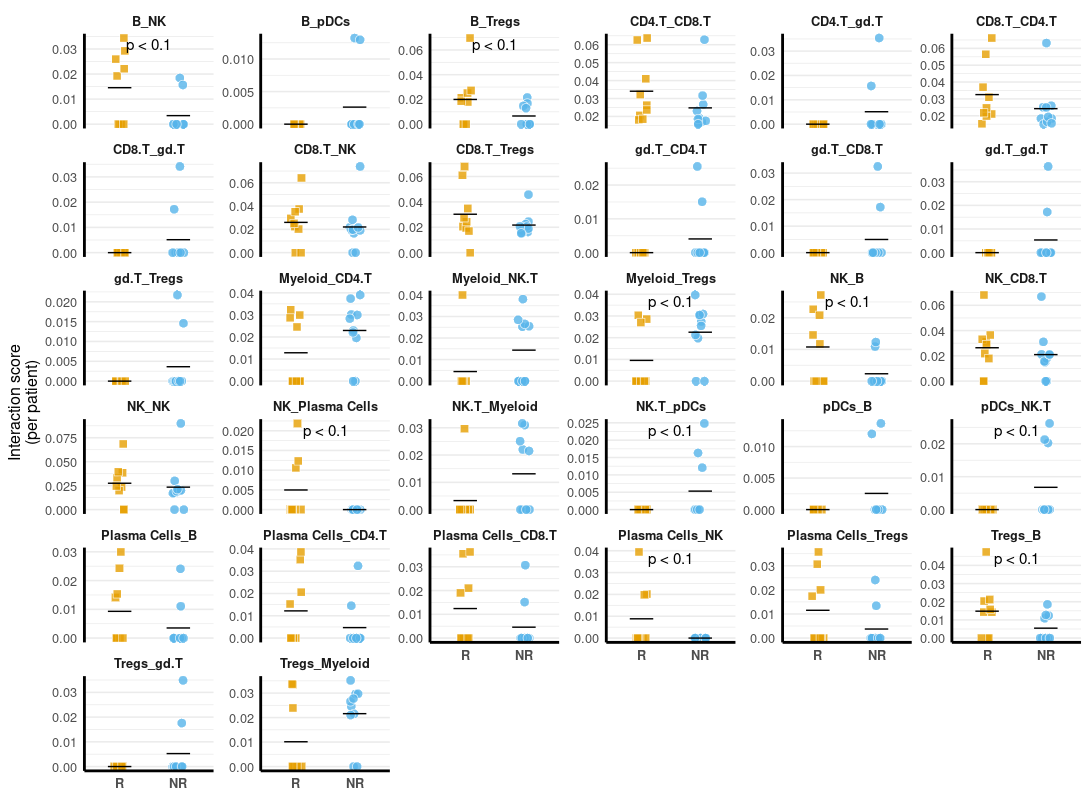


**a.** Cell proportions for the subset of patients where matched pre- and post-ICI samples were available.

**b.** Fraction of T cell subsets – defined as the number of cells in that subset divided by the total number of T cells in that sample – was compared across R and NR pre- and post-ICI groups. Th2 = CD4+ T helper 2, Th1 = CD4+ T helper 1, CD8T.Eff = CD8+ Effector T cell, CD8T.pEx = CD8+ T progenitor exhausted T cell, gdT = gamma delta T cell, MAIT = mucosal associated invariant T, Treg = regulatory T cell, CD8T.Exh = Exhausted CD8+ T cell.

**c.** Fraction of T cell subsets for patients where matched pre- and post-ICI samples were available.

**d.** Myeloid cell subsets in R and NR pre- and post-ICI groups.

**e.** Interaction frequencies for cell type pairs with R and NR differences with p-values between 0.05 and 0.25 (trending but less significant than those in Fig. 1e).

In all matched tests, a paired Wilcoxon test was used to determine significance. For non-matched two group comparisons, a Wilcoxon rank-sum test was used. For comparisons with more than two groups, Kruskal-Wallis followed by Dunn’s post-hoc test was used.

**Figure S3: Survival of Mixed Interaction Expression in Bulk RNA-seq**

**a.**  **
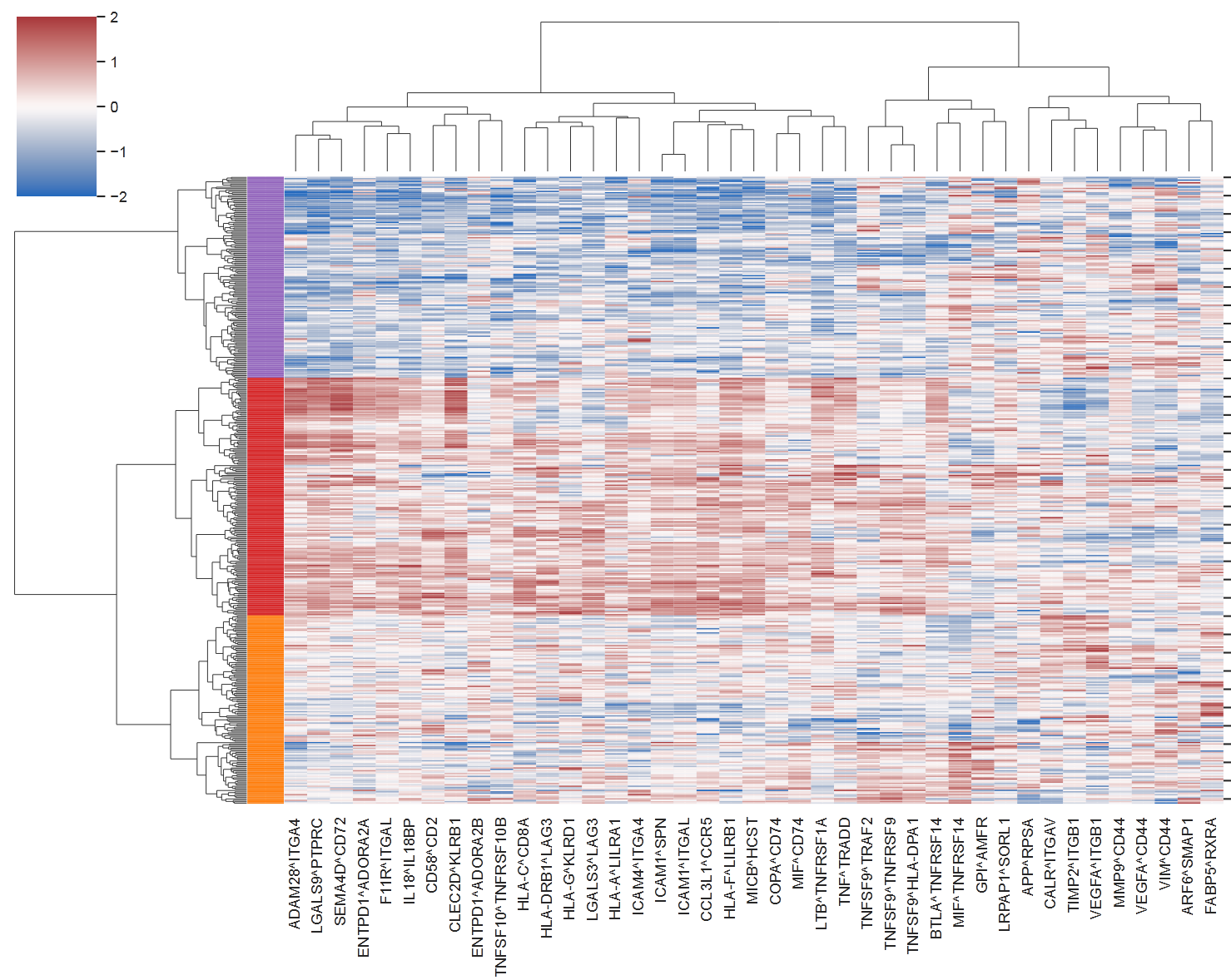
**

**b.**
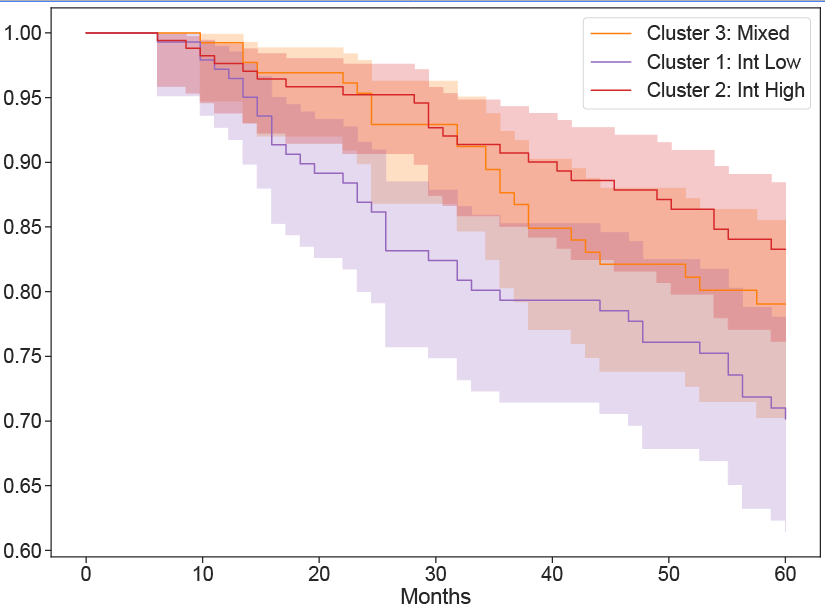


**a.** Average of z-scored expression data for LR pairs for melanoma patients, as shown in Fig. 4a, with coloring matching the three groups in panel b.

**b.** Survival for all three clusters. Mixed phenotype (Cluster 3: Orange) shows similar trends as interaction high (Cluster 2: Red).

**Figure S4: Pre- to Post-Treatment Comparison of Cell Abundance and Interaction Frequency**

**a.**

**
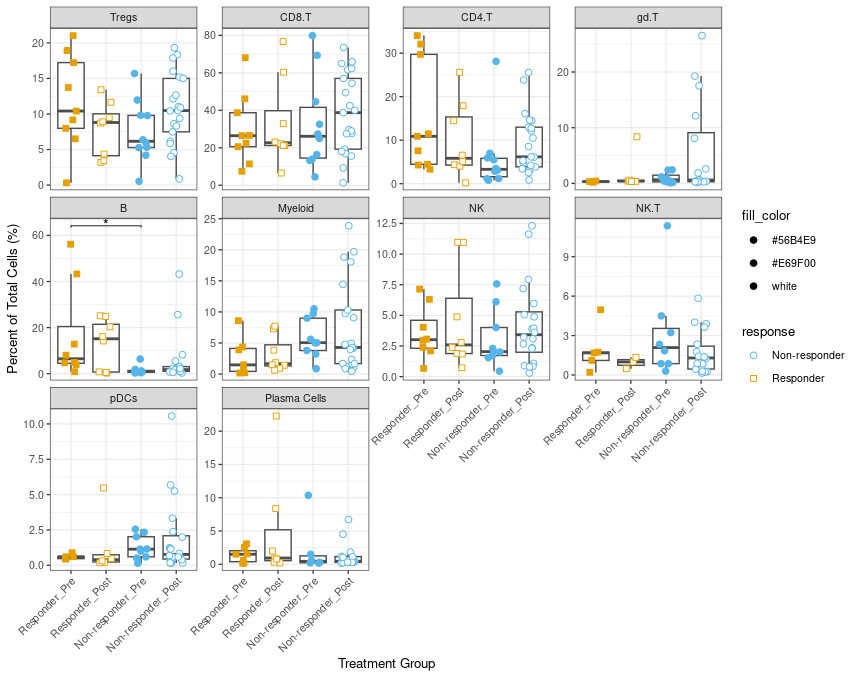
**

**b.**


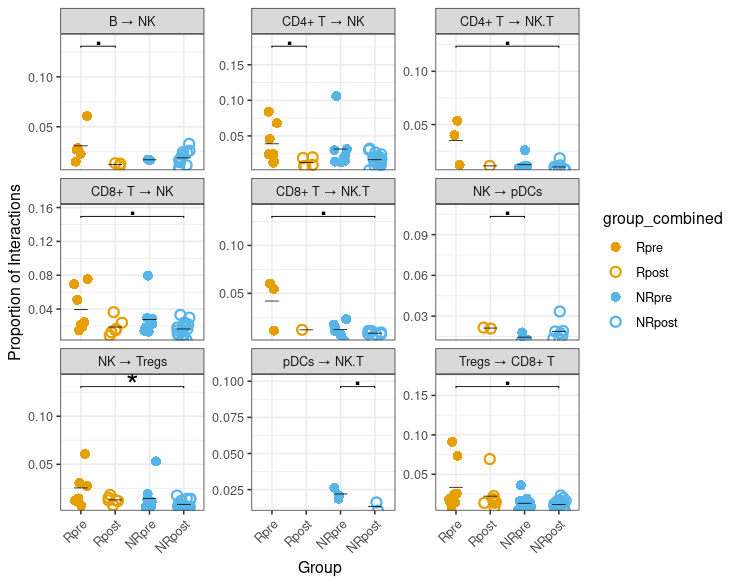


**a.** Cell type proportions pre-post in R and NR

**b.** Unique interaction frequencies pre- post in R and NR

In a, b, * denotes statistical significance. .p<0.1, *p<0.05, **p<0.01. Statistical significance was determined by a Kruskal-Wallis test followed by Dunn’s post-hoc test. The median is indicated by a colored line.

**Figure S5: Jerby-Arnon Additional Cell Type Proportion and Interaction Frequency Comparisons**

**a.**

**
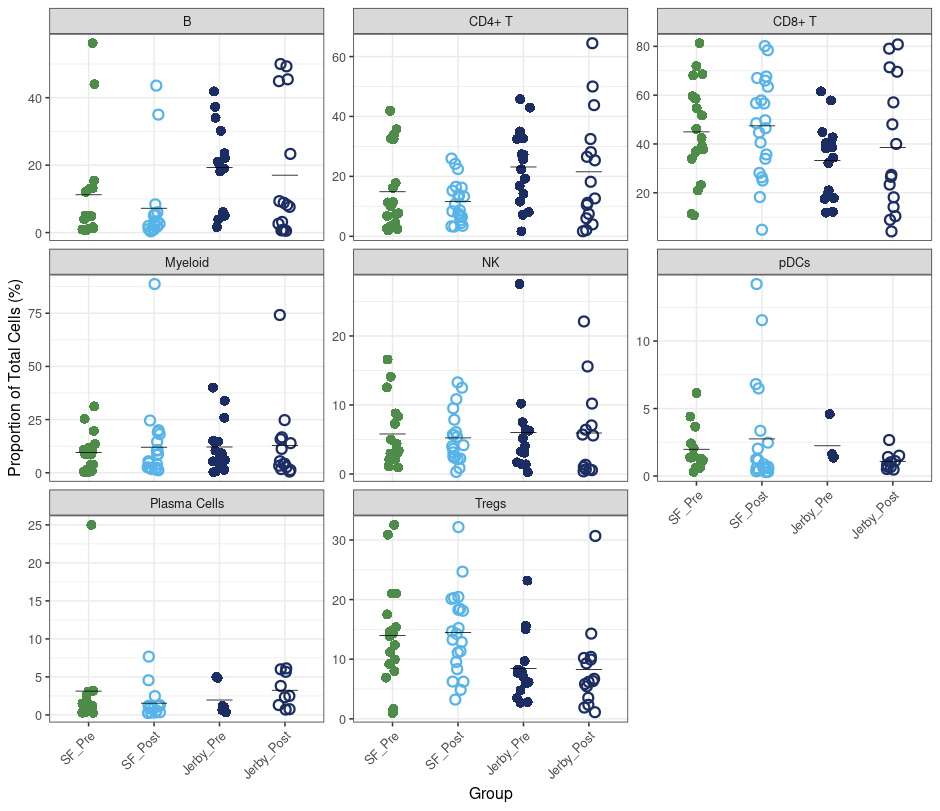
**

**b.

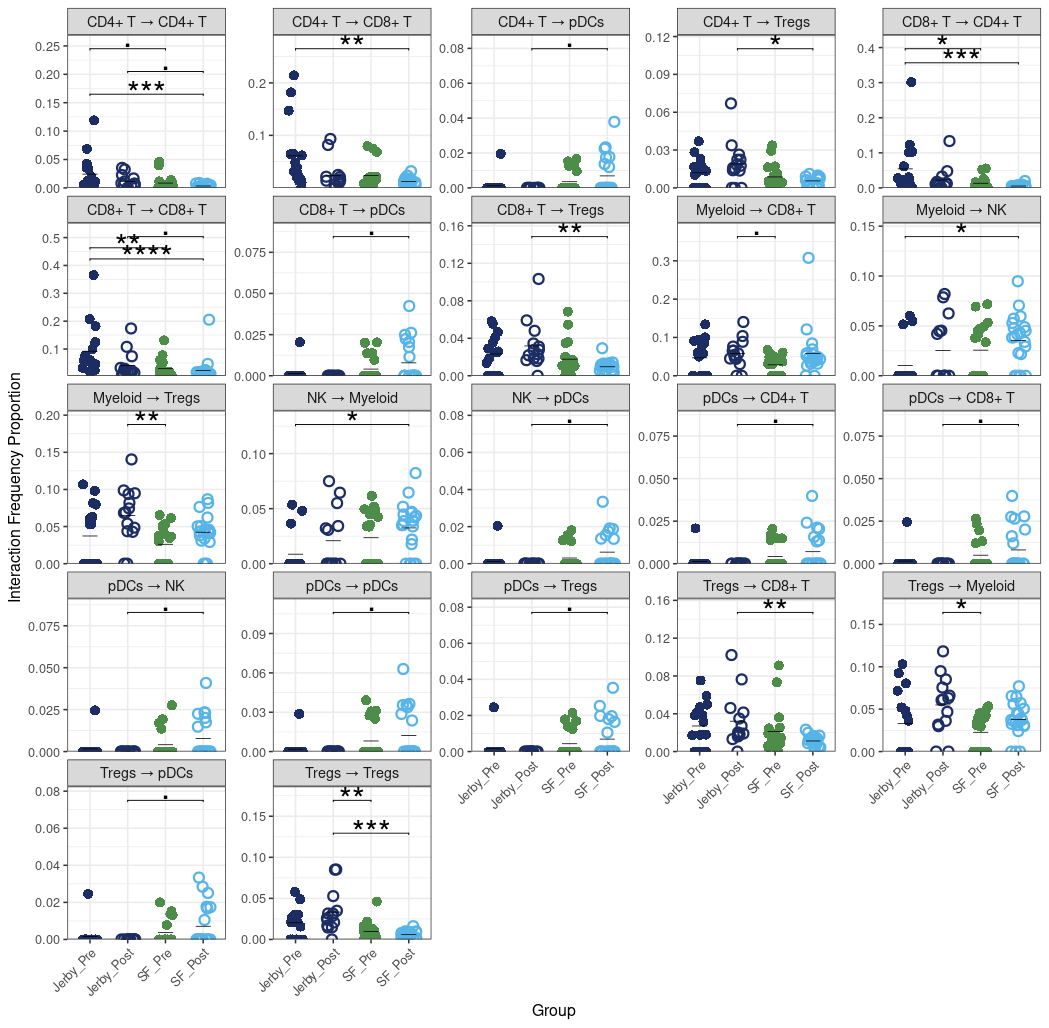
**

**a.** Coarse cell type proportions compared in all pre-treatment patients compared to post-NR in Jerby-Arnon data.

**b.** Univariate comparison of interaction frequency in both Jerby-Arnon and Sade-Feldman pre-treatment patients (all, no response stratification) and post-treatment NR.

**Supplemental Information References:**

1. T cytotoxic cells gene expression markers | PanglaoDB. Accessed May 29, 2025. https://www.panglaodb.se/markers.html?cell_type=%27T%20cytotoxic%20cells%27

2. Jerby-Arnon L, Shah P, Cuoco MS, et al. A Cancer Cell Program Promotes T Cell Exclusion and Resistance to Checkpoint Blockade. *Cell*. 2018;175(4):984-997.e24. doi:10.1016/j.cell.2018.09.006

3. Sade-Feldman M, Yizhak K, Bjorgaard SL, et al. Defining T Cell States Associated with Response to Checkpoint Immunotherapy in Melanoma. *Cell*. 2018;175(4):998-1013.e20. doi:10.1016/j.cell.2018.10.038

4. Zhao H, Li CC, Pardo J, et al. A Novel E3 Ubiquitin Ligase TRAC-1 Positively Regulates T Cell Activation. *J Immunol*. 2005;174(9):5288-5297. doi:10.4049/jimmunol.174.9.5288

5. Bio-Rad. The T Cell Marker, CD3 Antigen & Antibodies: Mini-review. Bio-Rad. Accessed May 29, 2025. https://www.bio-rad-antibodies.com/minireview-cd3-antibody.html

6. UniProt - P07766 CD3E Human. UniProt. Accessed May 29, 2025. https://www.uniprot.org/uniprotkb/P07766/entry

7. T helper cells gene expression markers | PanglaoDB. Accessed May 29, 2025. https://www.panglaodb.se/markers.html?cell_type=%27T%20helper%20cells%27

8. Imai T, Nagira M, Takagi S, et al. Selective recruitment of CCR4-bearing Th2 cells toward antigen-presenting cells by the CC chemokines thymus and activation-regulated chemokine and macrophage-derived chemokine. *Int Immunol*. 1999;11(1):81-88. doi:10.1093/intimm/11.1.81

9. Esensten JH, Helou YA, Chopra G, Weiss A, Bluestone JA. CD28 Costimulation: From Mechanism to Therapy. *Immunity*. 2016;44(5):973-988. doi:10.1016/j.immuni.2016.04.020

10. Bao K, Reinhardt RL. The differential expression of IL-4 and IL-13 and its impact on type-2 Immunity. *Cytokine*. 2015;75(1):25-37. doi:10.1016/j.cyto.2015.05.008

11. Hori S. FOXP3 as a master regulator of Treg cells. *Nat Rev Immunol*. 2021;21(10):618-619. doi:10.1038/s41577-021-00598-9

12. B cells gene expression markers | PanglaoDB. Accessed May 29, 2025. https://www.panglaodb.se/markers.html?cell_type=%27B%20cells%27

13. Vaughn SE, Foley C, Lu X, et al. Lupus risk variants in the PXK locus alter B-cell receptor internalization. *Front Genet*. 2015;5:450. doi:10.3389/fgene.2014.00450

14. PubChem. HLA-DRA - major histocompatibility complex, class II, DR alpha (human). Accessed May 29, 2025. https://pubchem.ncbi.nlm.nih.gov/gene/HLA-DRA/human

15. T regulatory cells gene expression markers | PanglaoDB. Accessed May 29, 2025. https://www.panglaodb.se/markers.html?cell_type=%27T%20regulatory%20cells%27

16. Polak K, Marchal P, Taroni C, et al. CD4+ regulatory T cells lacking Helios and Eos. *Biochem Biophys Res Commun*. 2023;674:83-89. doi:10.1016/j.bbrc.2023.06.087

17. Hinterbrandner M, Rubino V, Stoll C, et al. Tnfrsf4-expressing regulatory T cells promote immune escape of chronic myeloid leukemia stem cells. *JCI Insight*. 2021;6(23):e151797. doi:10.1172/jci.insight.151797

18. NK cells gene expression markers | PanglaoDB. Accessed May 29, 2025. https://www.panglaodb.se/markers.html?cell_type=%27NK%20cells%27

19. Mahaweni NM, Olieslagers TI, Rivas IO, et al. A comprehensive overview of FCGR3A gene variability by full-length gene sequencing including the identification of V158F polymorphism. *Sci Rep*. 2018;8(1):15983. doi:10.1038/s41598-018-34258-1

20. Huang LP, Lyu SC, Clayberger C, Krensky AM. Granulysin-Mediated Tumor Rejection in Transgenic Mice. *J Immunol Baltim Md 1950*. 2007;178(1):77-84. doi:10.4049/jimmunol.178.1.77

21. Lanier LL, Chang C, Spits H, Phillips JH. Expression of cytoplasmic CD3 epsilon proteins in activated human adult natural killer (NK) cells and CD3 gamma, delta, epsilon complexes in fetal NK cells. Implications for the relationship of NK and T lymphocytes. *J Immunol Baltim Md 1950*. 1992;149(6):1876-1880.

22. Liu X, Zhu Z, Wang X. Specificity and function of T cell subset identities using single-cell sequencing. *Clin Transl Discov*. 2023;3(3):e199. doi:10.1002/ctd2.199

23. Travaglini KJ, Nabhan AN, Penland L, et al. A molecular cell atlas of the human lung from single-cell RNA sequencing. *Nature*. 2020;587(7835):619-625. doi:10.1038/s41586-020-2922-4

24. Zhao P, Zou J, Zhou F, et al. Immune features of COVID-19 convalescent individuals revealed by a single-cell RNA sequencing. *Int Immunopharmacol*. 2022;108:108767. doi:10.1016/j.intimp.2022.108767

25. Peng L, Jin X, Li B ya, et al. Integrating single-cell RNA sequencing with spatial transcriptomics reveals immune landscape for interstitial cystitis. *Signal Transduct Target Ther*. 2022;7(1):161. doi:10.1038/s41392-022-00962-8

26. Bost P, De Sanctis F, Canè S, et al. Deciphering the state of immune silence in fatal COVID-19 patients. *Nat Commun*. 2021;12(1):1428. doi:10.1038/s41467-021-21702-6

27. Kim N, Kim HK, Lee K, et al. Single-cell RNA sequencing demonstrates the molecular and cellular reprogramming of metastatic lung adenocarcinoma. *Nat Commun*. 2020;11(1):2285. doi:10.1038/s41467-020-16164-1

28. Monocytes gene expression markers | PanglaoDB. Accessed May 29, 2025. https://www.panglaodb.se/markers.html?cell_type=%27Monocytes%27

29. Macrophages gene expression markers | PanglaoDB. Accessed May 29, 2025. https://www.panglaodb.se/markers.html?cell_type=%27Macrophages%27

30. Gu Z, Wang L, Dong Q, et al. Aberrant LYZ expression in tumor cells serves as the potential biomarker and target for HCC and promotes tumor progression via csGRP78. *Proc Natl Acad Sci*. 2023;120(29):e2215744120. doi:10.1073/pnas.2215744120

31. Plasma cells gene expression markers | PanglaoDB. Accessed May 29, 2025. https://www.panglaodb.se/markers.html?cell_type=%27Plasma%20cells%27

32. Suimon Y, Kase S, Miura I, Ishijima K, Ishida S. Alteration of Cell Surface Markers CD38 and CD138 in Lymphoproliferative Disorders in the Ocular Adnexa. *Anticancer Res*. 2020;40(4):2019-2023. doi:10.21873/anticanres.14158

33. Tanaka T, Ichimura K, Sato Y, et al. Frequent downregulation or loss of CD79a expression in plasma cell myelomas: potential clue for diagnosis. *Pathol Int*. 2009;59(11):804-808. doi:10.1111/j.1440-1827.2009.02448.x

34. Wang K, Wei G, Liu D. CD19: a biomarker for B cell development, lymphoma diagnosis and therapy. *Exp Hematol Oncol*. 2012;1(1):36. doi:10.1186/2162-3619-1-36

35. CD19 protein expression summary - The Human Protein Atlas. Accessed May 29, 2025. https://www.proteinatlas.org/ENSG00000177455-CD19

36. Pavlasova G, Mraz M. The regulation and function of CD20: an “enigma” of B-cell biology and targeted therapy. *Haematologica*. 2020;105(6):1494-1506. doi:10.3324/haematol.2019.243543

37. Khaled Y, Fondaw M, Balls J, Smith T, Solh M. Plasma Cell CD20 Expression: Primary Aberrant Expression or Receptor Up-Regulation. *Biol Blood Marrow Transplant*. 2013;19(2):S235. doi:10.1016/j.bbmt.2012.11.297

38. Plasmacytoid dendritic cells gene expression markers | PanglaoDB. Accessed May 29, 2025. https://www.panglaodb.se/markers.html?cell_type=%27Plasmacytoid%20dendritic%20cells%27

39. LILRA4 leukocyte immunoglobulin like receptor A4 [Homo sapiens (human)] - Gene - NCBI. Accessed May 29, 2025. https://www.ncbi.nlm.nih.gov/gene/23547

40. Yasaka K, Yamazaki T, Sato H, et al. Phospholipase D4 as a signature of toll-like receptor 7 or 9 signaling is expressed on blastic T-bet + B cells in systemic lupus erythematosus. *Arthritis Res Ther*. 2023;25:200. doi:10.1186/s13075-023-03186-5

41. PLD4 protein expression summary - The Human Protein Atlas. Accessed May 29, 2025. https://www.proteinatlas.org/ENSG00000166428-PLD4?utm_source=chatgpt.com

42. Mullan KA, de Vrij N, Valkiers S, Meysman P. Current annotation strategies for T cell phenotyping of single-cell RNA-seq data. *Front Immunol*. 2023;14:1306169. doi:10.3389/fimmu.2023.1306169

43. Fontenot JD, Gavin MA, Rudensky AY. Foxp3 programs the development and function of CD4+CD25+ regulatory T cells. *Nat Immunol*. 2003;4(4):330-336. doi:10.1038/ni904

44. Beltra JC, Manne S, Abdel-Hakeem MS, et al. Developmental Relationships of Four Exhausted CD8+ T Cell Subsets Reveals Underlying Transcriptional and Epigenetic Landscape Control Mechanisms. *Immunity*. 2020;52(5):825-841.e8. doi:10.1016/j.immuni.2020.04.014

45. Khan O, Giles JR, McDonald S, et al. TOX transcriptionally and epigenetically programs CD8+ T cell exhaustion. *Nature*. 2019;571(7764):211-218. doi:10.1038/s41586-019-1325-x

46. Szabo PA, Levitin HM, Miron M, et al. Single-cell transcriptomics of human T cells reveals tissue and activation signatures in health and disease. *Nat Commun*. 2019;10(1):4706. doi:10.1038/s41467-019-12464-3

47. Pappalardo JL, Zhang L, Pecsok MK, et al. Transcriptomic and clonal characterization of T cells in the human central nervous system. *Sci Immunol*. 2020;5(51):eabb8786. doi:10.1126/sciimmunol.abb8786

48. Hu Y, Hu Q, Li Y, et al. γδ T cells: origin and fate, subsets, diseases and immunotherapy. *Signal Transduct Target Ther*. 2023;8(1):434. doi:10.1038/s41392-023-01653-8

49. Wong EB, Gold MC, Meermeier EW, et al. TRAV1-2+ CD8+ T-cells including oligoconal expansions of MAIT cells are enriched in the airways in human tuberculosis. *Commun Biol*. 2019;2(1). doi:10.1038/s42003-019-0442-2

50. Terpstra ML, Remmerswaal EBM, van der Bom-Baylon ND, et al. Tissue-resident mucosal-associated invariant T (MAIT) cells in the human kidney represent a functionally distinct subset. *Eur J Immunol*. 2020;50(11):1783-1797. doi:10.1002/eji.202048644

51. Shan Q, Li X, Chen X, et al. Tcf1 and Lef1 provide constant supervision to mature CD8+ T cell identity and function by organizing genomic architecture. *Nat Commun*. 2021;12(1):5863. doi:10.1038/s41467-021-26159-1

52. Steinke FC, Yu S, Zhou X, et al. TCF-1 and LEF-1 act upstream of Th-POK to promote the CD4(+) T cell fate and interact with Runx3 to silence Cd4 in CD8(+) T cells. *Nat Immunol*. 2014;15(7):646-656. doi:10.1038/ni.2897

53. Kaminskiy Y, Kuznetsova V, Kudriaeva A, Zmievskaya E, Bulatov E. Neglected, yet significant role of FOXP1 in T-cell quiescence, differentiation and exhaustion. *Front Immunol*. 2022;13:971045. doi:10.3389/fimmu.2022.971045

54. Galletti G, De Simone G, Mazza EMC, et al. Two subsets of stem-like CD8+ memory T cell progenitors with distinct fate commitments in humans. *Nat Immunol*. 2020;21(12):1552-1562. doi:10.1038/s41590-020-0791-5

55. Spolski R, Leonard WJ. IL-21 and T follicular helper cells. *Int Immunol*. 2010;22(1):7-12. doi:10.1093/intimm/dxp112

56. Zhang J, Roberts AI, Liu C, et al. A novel subset of helper T cells promotes immune responses by secreting GM-CSF. *Cell Death Differ*. 2013;20(12):1731-1741. doi:10.1038/cdd.2013.130

57. Yi K, Jo S, Song W, et al. Analysis of Single-Cell Transcriptome and Surface Protein Expression in Ankylosing Spondylitis Identifies OX40-Positive and Glucocorticoid-Induced Tumor Necrosis Factor Receptor–Positive Pathogenic Th17 Cells. *Arthritis Rheumatol*. 2023;75(7):1176-1186. doi:10.1002/art.42476

58. Guimarães GR, Maklouf GR, Teixeira CE, et al. Single-cell resolution characterization of myeloid-derived cell states with implication in cancer outcome. *Nat Commun*. 2024;15(1):5694. doi:10.1038/s41467-024-49916-4

59. Cheng S, Li Z, Gao R, et al. A pan-cancer single-cell transcriptional atlas of tumor infiltrating myeloid cells. *Cell*. 2021;184(3):792-809.e23. doi:10.1016/j.cell.2021.01.010

60. Wang J, Zhu N, Su X, Gao Y, Yang R. Novel tumor-associated macrophage populations and subpopulations by single cell RNA sequencing. *Front Immunol*. 2024;14:1264774. doi:10.3389/fimmu.2023.1264774

61. Revel M, Sautès-Fridman C, Fridman WH, Roumenina LT. C1q+ macrophages: passengers or drivers of cancer progression. *Trends Cancer*. 2022;8(7):517-526. doi:10.1016/j.trecan.2022.02.006

62. Dang MT, Gonzalez MV, Gaonkar KS, et al. Macrophages in SHH subgroup medulloblastoma display dynamic heterogeneity that varies with treatment modality. *Cell Rep*. 2021;34(13):108917. doi:10.1016/j.celrep.2021.108917

63. Zhang L, Li Z, Skrzypczynska KM, et al. Single-Cell Analyses Inform Mechanisms of Myeloid-Targeted Therapies in Colon Cancer. *Cell*. 2020;181(2):442-459.e29. doi:10.1016/j.cell.2020.03.048
